# Targeting PRMT5 alleviates diabetic cardiomyopathy: mechanisms involve Ndufs2 R118 dimethylation-mediated complex I protection and serum PRMT5 as a biomarker

**DOI:** 10.1186/s10020-026-01594-2

**Published:** 2026-08-13

**Authors:** Sidong Cai, Mengqi Su, Jing Li, Yang Yu, Min Wu, Mingya Liu, Run Wang, Jie Huang, Qingxia Lai, Cong Chen, Kai-Hang Yiu

**Affiliations:** 1 Department of Medicine, Division of Cardiology, The University of Hong Kong-Shenzhen Hospital, Shenzhen, China; 2 Department of Ultrasound, Yantian District People’s Hospital, Shenzhen, China; 3 Shenzhen University Health Science Center, Shenzhen, China; 4 Department of Cardiology, The University of Hong Kong, Queen Mary Hospital, Hong Kong, China

**Keywords:** PRMT5, Ndufs2, Symmetric di-methylation (SDMA), Complex I, Diabetic cardiomyopathy (DCM)

## Abstract

**Background:**

Diabetic cardiomyopathy (DCM) is defined by progressive cardiac dysfunction driven by myocardial ATP depletion, where mitochondrial electron transport chain (ETC) Complex I dysfunction acts as a critical factor. Protein arginine methyltransferase 5 (PRMT5) maintains cardiac homeostasis, yet its function in DCM and association with Complex I remain elusive. This study aimed to investigate the function, key substrate, and mechanism of PRMT5 in DCM, and evaluate serum PRMT5 as a potential biomarker for DCM.

**Methods:**

Gain- and loss-of-function experiments of PRMT5 were conducted to explore the function of PRMT5 in DCM. IP-MS proteomics and post-translational modification analysis were applied to identify the substrates of PRMT5 and their modified sites. By employing ELISA assay, serum levels of PRMT5, lactate, and lactate/pyruvate (L/P) ratio were measured in DCM patients, and the association between serum PRMT5 level and hospital readmission risk was analyzed.

**Results:**

PRMT5 overexpression protected against DCM pathogenesis, while its knockdown or inhibition exacerbated disease progression. PRMT5 mediated symmetric di-methylation (SDMA) of Ndufs2 at R118 to prevent its ubiquitin-proteasomal degradation and preserve mitochondrial Complex I function. DCM patients had significantly lower serum PRMT5, which correlated positively with serum lactate and L/P ratio and was associated with higher hospital readmission risk.

**Conclusions:**

Our findings unveil a novel PRMT5/Ndufs2 regulatory axis where arginine methylation maintains bioenergetic capacity of Complex I through methylation-ubiquitination crosstalk. PRMT5 shows potential as a therapeutic target and biomarker for DCM, which may aid the establishment of innovative diagnostic and treatment frameworks for DCM pending further translational validation.

**Graphical Abstract:**

**Figure:**
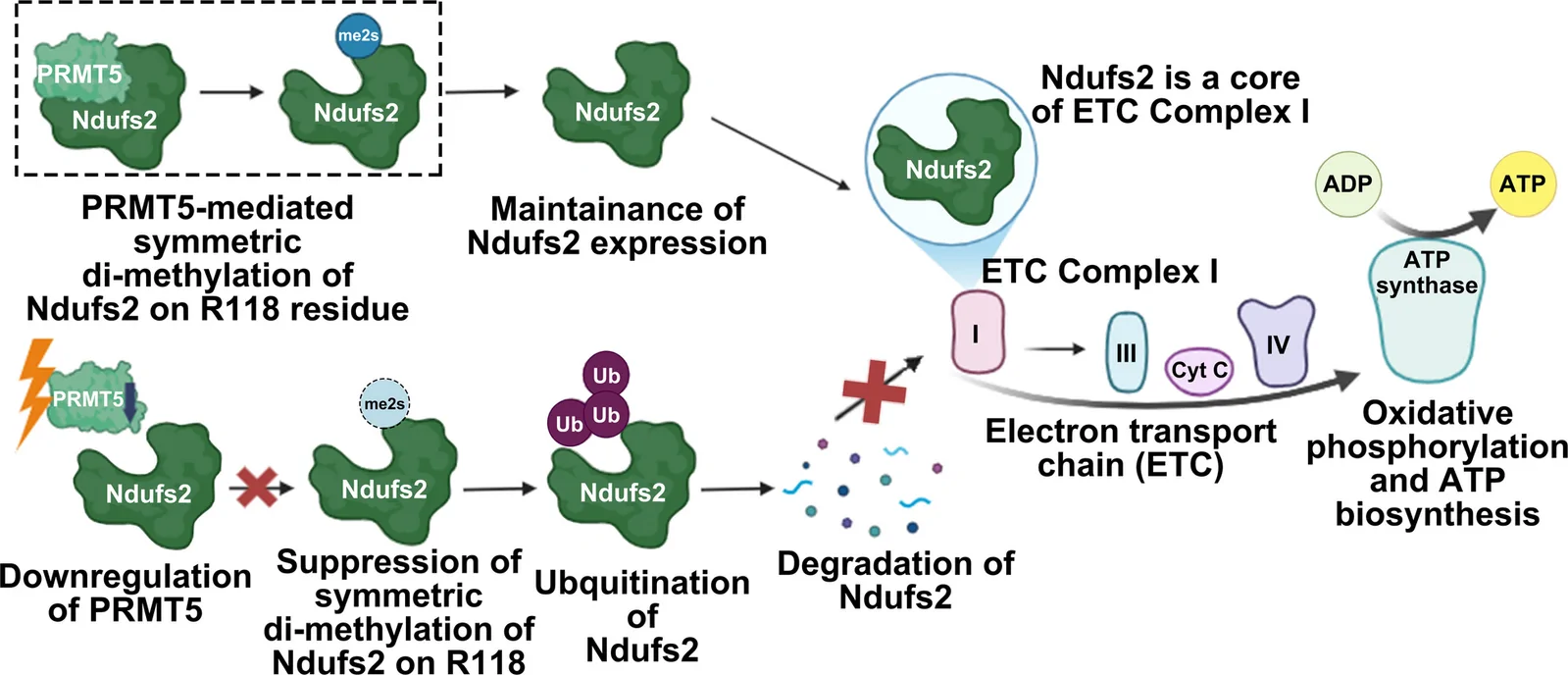

**Supplementary Information:**

The online version contains supplementary material available at https://doi.org/10.1186/s10020-026-01594-2.

## Background

Diabetic cardiomyopathy (DCM), a pathological consequence of diabetes mellitus, is characterized by progressive cardiac dysfunction leading to heart failure (Dillmann 2019; Jankauskas et al. 2021; Yu et al. 2025). Central to its pathogenesis is chronic myocardial ATP depletion, which disrupts cardiac contractility and compromises systemic energy supply (Chang et al. 2022). Despite improved therapeutic outcomes reducing mortality, persistent patient disability highlights unresolved mechanistic complexity, emphasizing the need to identify new regulatory factors and their mechanisms in DCM. To date, multiple mechanisms have been reported to contribute to diabetic cardiac ATP depletion (Kiss and Szabó 2005; Roussel et al. 2015). Emerging evidence implicates mitochondrial dysfunction as a key contributor to ATP deficiency, particularly through impaired oxidative phosphorylation (OXPHOS) via electron transport chain (ETC) Complexes I-V dysfunction (Bhullar and Dhalla 2023). While pharmacological enhancement of rate-limiting Complex I shows preclinical promise for restoring cardiac bioenergetics, clinical strategies remain lacking.

Ndufs2 (NADH dehydrogenase [ubiquinone] iron-sulfur protein 2) serves as the structural and functional core subunit of mitochondrial Complex I, mediated NADH-driven electron transfer (Dunham-Snary et al. 2019; Ngu et al. 2012). Previous studies demonstrated that Ndufs2 knockdown in pulmonary artery smooth muscle cells reduces Complex I activity by 62% (Bandara et al. 2021), and its disruption impairs Complex I specific respiration, glycolytic capacity, ATP pool and cell-membrane integrity (Kowaltowski et al. 2009). Although the association between Complex I dysfunction and diabetic cardiomyopathy (DCM) is well established, and Ndufs2 plays a critical role in regulating Complex I activity, the pathophysiological relevance of this core subunit to DCM remains underexplored. Specifically, how Ndufs2 expression and function are post-translationally regulated in the diabetic heart, and whether this regulation contributes to Complex I dysfunction and ATP depletion, remains unclear.

Protein arginine methyltransferase 5 (PRMT5) is the major enzyme mediating arginine symmetric di-methylation (SDMA) (Kim and Ronai 2020; Wang et al. 2014). By transferring methyl groups from S-adenosylmethionine (SAM) to arginine residues, PRMT5 regulates the structure and function of its substrate proteins (Lorton and Shechter 2019). Cardiac tissue exhibits particularly high PRMT5 expression, with emerging evidence implicating its SDMA activity in cardiovascular pathophysiology. It is reported that specific PRMT5 ablation induces diastolic dysfunction and cardiomyopathy in mice, whereas inhibiting its SDMA activity exacerbates cardiomyocyte hypertrophy (Li et al. 2021; Chen et al. 2014). Notably, PRMT5 genetic variants correlate with hypertrophic cardiomyopathy progression, with frequent mutations in patient myocardial biopsies (Cui et al. 2022). Therefore, the role of PRMT5 and its SDMA activity may function as promising therapeutic targets in DCM. Nonetheless, PRMT5-mediated epigenetic regulation crosstalk with mitochondrial ETC function is unelucidated. Whether PRMT5 directly targets Complex I subunit Ndufs2 via SDMA modification, and how this modification modulates Complex I activity and myocardial bioenergetics in DCM, has not been addressed.

Based on these knowledge gaps, we hypothesize that PRMT5 protects against DCM by SDMA modification of Ndufs2, thereby inhibiting Ndufs2 degradation, preserving Complex I integrity, and maintaining ATP production. Thus, the present study aims to investigate the role of PRMT5 in DCM, focuses on Ndufs2 as a potential substrate, and uncovers mechanisms linking PRMT5-mediated Ndufs2 SDMA modification to mitochondrial Complex I activity and myocardial bioenergetics.

## Methods

### Primary cell culture

Neonatal rat cardiomyocytes (NRCMs) were isolated and cultured as previously described (Ravi et al. 2021). NRCMs were seeded into flasks/plates in growth DMEM containing 10% FBS, 1% penicillin–streptomycin and 0.1 mM 5-bromodeoxyuridine (5-BrdU) and cultured for 24 h prior to experiments. To ensure experimental reliability, at least 1.5–2.0 × 10^6 NRCMs were isolated from each rat heart, α-actin staining confirmed cardiomyocyte purity > 90%, and a minimum cell confluency of 70% was required before subsequent experimental interventions.

### Animal procedures

#### Statement for ethics approval and animal welfare compliance

All animal experiments were performed in strict accordance with institutional guidelines and approved by the Medical Ethics Committee of the University of Hong Kong-Shenzhen Hospital (Approval No.: [2021] 066). All procedures complied with the Directive 2010/63/EU and the NIH Guide for the Care and Use of Laboratory Animals (8th edition) (National Research Council (US) Committee for the Update of the Guide for the Care and Use of Laboratory Animals 2011; Donovan and Brown 2013). Following the 3R principles (Replacement, Reduction, Refinement), 6 mice were used per group to achieve statistical significance while minimizing animal usage.

#### Statement for employment of anaesthetic agents

All anaesthetics used for echocardiography, intramyocardial adenovirus injection, and euthanasia are described below with their generic names, doses, administration routes and frequencies, in accordance with animal welfare guidelines and to ensure experimental reproducibility.

1. For echocardiographic measurements: Mice were anesthetized with isoflurane. Anaesthesia was induced with 3.0–3.5% isoflurane in oxygen for 2–3 min until loss of righting reflex and a stable respiratory rate (60–80 breaths/min). During measurements, 1.0–1.5% isoflurane was maintained via facemask with an oxygen flow of 0.8–1.0 L/min.
2. For intramyocardial adenovirus delivery: Mice were anesthetized with 3.0–3.5% isoflurane for induction and maintained with 1.0–1.5% isoflurane under tracheal intubation and mechanical ventilation. For analgesia, buprenorphine (0.05–0.1 mg/kg) was administered intraperitoneally 30 min before surgery, then every 12 h for 48 h postoperatively.
3. For euthanasia: Mice were deeply anesthetized with 3.0–3.5% isoflurane until respiratory arrest (≥ 30 s) and absent heartbeat (≥ 1 min) were confirmed, followed by cervical dislocation.

#### Feeding and allocations

Specific pathogen‑free (SPF) male C57BL/6 mice (18–22 g) were obtained from Zhuhai BesTest Bio-Tech Co., Ltd. (Zhuhai, China). Mice were housed in an SPF facility (21–23 °C, 12 h light/dark cycle) with free access to standard chow and water, and acclimatized for 1 week before experiments. Animals were randomly assigned by an investigator blinded to group allocation using a random number table. Twelve mice were equally divided into two groups for DCM induction: control (CON) and high‑fat diet/streptozotocin (HFD/STZ) groups. Twenty‑four mice were randomized into four groups to assess the role of PRMT5 in DCM: Ad‑vector + CON, Ad‑PRMT5 + CON, Ad‑vector + HFD/STZ, and Ad‑PRMT5 + HFD/STZ.

#### Establishment of DCM

Diabetes was confirmed at week 24 according to WHO criteria: fasting blood glucose (FBG) ≥ 7.0 mmol/L and glycated hemoglobin (HbA1c) ≥ 6.5%. STZ was dissolved in 0.1 mol/L citrate buffer (pH 4.4) following the manufacturer’s instructions. High-fat diet (HFD, D12492; 20% carbohydrate, 20% protein, 60% fat, kcal%) and standard chow (D12450B; 70% carbohydrate, 20% protein, 10% fat, kcal%) were obtained from Research Diets, Inc.

**Fig. 1:**
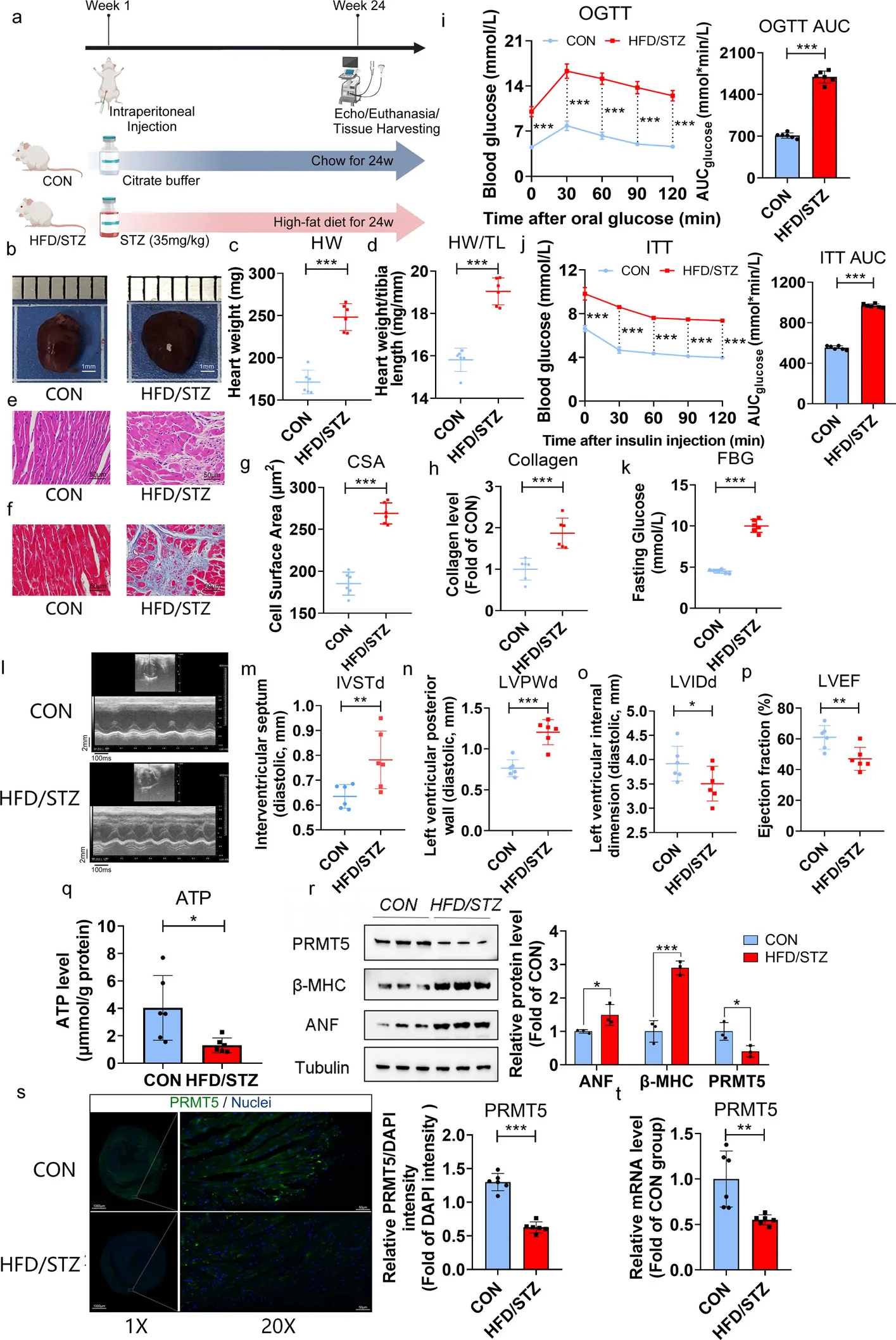
PRMT5 is downregulated in the DCM heart. (**a**) Schematic of the HFD/STZ-induced DCM mouse model. (**b**) Representative gross heart morphology. Scale bar = 1 mm. (**c**-**d**) Quantification of heart weight (HW; **c**) and HW/tibia length (TL) ratio (**d**). (**e**–**f**) Representative H&E (**e**) and Masson's trichrome (**f**) staining of heart sections. Scale bar = 50 μm. (**g**-**h**) Quantification of cardiomyocyte cross-sectional area (CSA) from H&E (**g**) and myocardial collagen content from Masson's staining (**h**). (**i**–**k**) Metabolic assessments: oral glucose tolerance test (OGTT) and AUC (**i**), insulin tolerance test (ITT) and AUC (**j**), and fasting blood glucose (FBG, k). (**l**) Representative M-mode echocardiographic images. Scale bar = 2 mm, time scale = 100 ms. (**m**–**p**) Echocardiographic parameters: IVSTd (m), LVPWd (n), LVIDd (**o**) and LVEF (**p**). (**q**) Myocardial ATP content measured by ELISA. (**r**) Western blot analysis of ANF, β-MHC and PRMT5. (**s**) Immunofluorescence staining of PRMT5 (green) with DAPI counterstain (blue). Scale bars: 1000 μm (left) and 50 μm (right). Data are presented by quantification of PRMT5 fluorescence intensity relative to DAPI. (t) qPCR analysis of PRMT5 mRNA level. Data are demonstrated by mean ± SD. *P* values by unpaired two-tailed Student's t-test versus CON group. *p < 0.05; **p < 0.01; ***p < 0.001, *n* = 6

To establish DCM model, mice in the HFD/STZ, Ad-vector + HFD/STZ, and Ad-PRMT5 + HFD/STZ groups were fed a HFD for 24 weeks and received a single intraperitoneal injection of STZ (35 mg/kg) on day 7. Mice in the CON, Ad-vector + CON, and Ad-PRMT5 + CON groups were fed standard chow and injected with an equal volume of vehicle citrate buffer. All mice were maintained under these conditions until echocardiographic analysis at week 24. The experimental timeline for DCM induction is shown in Fig. 1a.

#### Intramyocardial delivery of recombinant adenovirus encoding PRMT5 gene

Prior to adenovirus injection at Week 12, we performed pre-intervention cardiac phenotype validation via echocardiographic assessments in all mice. These analyses confirmed significant cardiac structural remodeling (slightly increased IVSTd and decreased LVIDd) and functional impairment (decreased LVEF) in mice with DCM, with no obvious abnormalities in the control mice, validating stable pre-intervention phenotypes (Supplementary Figure S1a-c).

Mice were divided into four groups: Ad‑vector + CON, Ad‑PRMT5 + CON, Ad‑vector + HFD/STZ, and Ad‑PRMT5 + HFD/STZ. At week 12, all mice were anesthetized with isoflurane and subjected to tracheal intubation and mechanical ventilation. Details of anesthesia are described in Sect. 2.2.2. For analgesia, buprenorphine (0.05–0.1 mg/kg) was administered intraperitoneally 30 min before surgery and every 12 h for 48 h postoperatively.

To expose the heart, a small left thoracotomy was performed at the 3rd to 4th intercostal space to expose the heart. Adenovirus (2 × 10^9 pfu in 200 μL, WZ Biosciences Inc.) was injected into 5–6 sites along the left ventricular wall using a 25‑gauge curved needle. Mice in the Ad‑PRMT5 groups received PRMT5‑expressing adenovirus, whereas those in the Ad‑vector groups were given empty vector (*n* = 6 per group). The injection depth was strictly controlled at 2–3 mm to avoid ventricular cavity penetration or virus leakage due to insufficient depth. The injection sites were evenly distributed over the anterior, lateral, and posterior walls of the left ventricle (1–2 sites per wall) to ensure uniform diffusion of the virus in myocardial tissue. A microsyringe was used for slow injection at a rate of 10 μL/min, and the needle was retained for 10 s after injection before slow withdrawal to minimize virus leakage along the needle tract.

Immediately after cardiac adenovirus injection, the wound was sutured and cleaned with sterile gauze soaked in 10% povidone-iodine solution, followed by 2–3 mg/kg body weight of gentamycin via intraperitoneal injection once daily for 3 consecutive days after intra-myocardial delivery of recombinant adenovirus. Mice were monitored daily for 7 days post-surgery to record survival status, body weight changes, and wound healing. Exclusion criteria were clearly defined as: (1) death within 24 h post-surgery (considered surgery-related mortality); (2) wound infection (local redness, suppuration) or uncontrolled bleeding; (3) > 20% body weight loss.

To validate the cardiac specificity of intramyocardial adenovirus delivery, we assessed PRMT5 mRNA expression across multiple organs (heart, liver, spleen, lung, and kidney) after Ad-PRMT5 or Ad-vector injection. Quantitative PCR analysis revealed that PRMT5 mRNA was significantly upregulated exclusively in the heart, with no statistically significant changes detected in other organs examined, confirming predominant cardiac transduction with minimal systemic off-target expression (Supplementary Figure S1d).

**Fig. 2:**
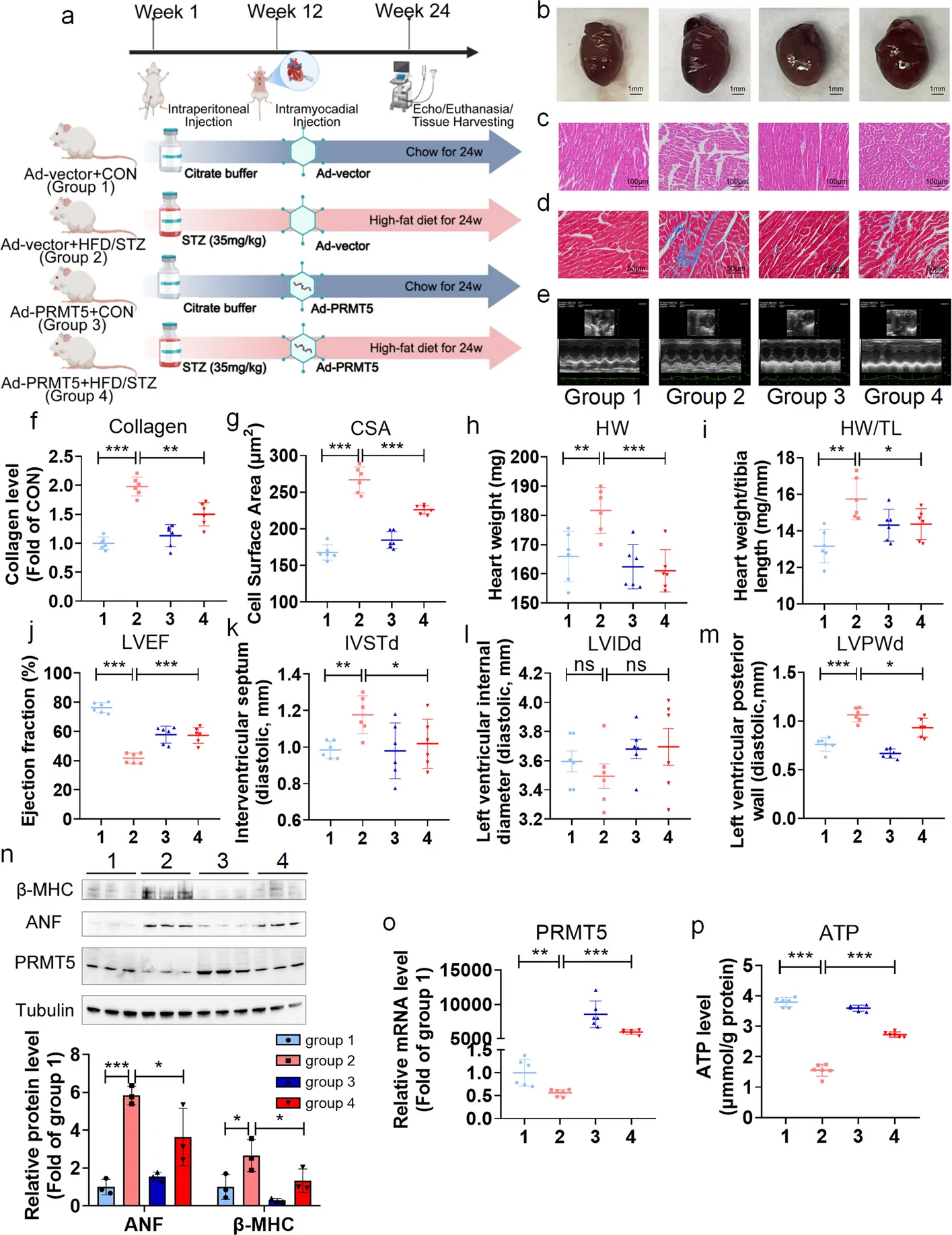
PRMT5 protects against DCM in vivo. Mice were intracardially injected with Ad-vector or Ad-PRMT5, followed by HFD/STZ induction. **a** Schematic of the experimental procedure in four designed groups: Ad-vector + CON, Ad-vector + HFD/STZ, Ad-PRMT5 + CON, and Ad-PRMT5 + HFD/STZ. **b** Representative gross heart morphology. Scale bar = 1 mm. (c-d) Representative H&E **c** and Masson's trichrome (**d**) staining of cardiac sections. Scale bar = 100 μm **c**, 50 μm **d**. **e** Representative M-mode echocardiographic images. Scale bar = 2 mm, time scale = 100 ms. (**f**–**g**) Quantification of collagen deposition (**f**) and CSA (**g**). **h**–**i** Quantification of HW (h) and HW/TL ratio (i). (**j**–**m**) Echocardiographic parameters: LVEF (**j**), IVSTd (**k**), LVIDd (**l**) and LVPWd (**m**). **n** Western blot analysis of ANF and β-MHC. **o** Myocardial ATP content measured by ELISA. **p** Cardiac PRMT5 mRNA level by qPCR. Data are presented as mean ± SD. P values by one-way ANOVA with Tukey's post-hoc test. *P < 0.05; **P < 0.01; ***P < 0.001, ns, no significance, *n* = 6

The schemas illustrating the timeline of the principal experimental procedures in mice are presented in Fig. 2a.

### Fasting blood glucose (FBG) and oral glucose tolerance test (OGTT)

Fasting blood glucose (FBG) was measured from the tail vein of 12-h-fasted mice. For oral glucose tolerance test (OGTT), fasted mice were gavaged with 20% glucose (2 g/kg body weight), and blood glucose was measured at 0, 30, 60, 90, and 120 min.

All glucose measurements were performed with a Roche glucometer calibrated with two-point solutions (4.4 mmol/L and 16.7 mmol/L) for accuracy. Each time point was measured in duplicate: the average was used if duplicates differed by ≤ 0.5 mmol/L; a third measurement was performed if the difference exceeded 0.5 mmol/L, and the average of the two closest values was adopted.

OGTT results were analyzed by calculating the area under the curve (AUC) and normalizing to the baseline (0 min) glucose level to eliminate individual baseline differences. Samples were considered invalid if glucose levels were outside the glucometer’s detection limit (< 1.1 mmol/L or > 33.3 mmol/L) and were retested after ≥ 7 days in the same mice.

### Insulin tolerance test (ITT)

The ITT was performed as follows: Mice were fasted for 6 h prior to the experiment, followed by intraperitoneal injection of insulin at a dose of 3 units/kg body weight. Plasma glucose was measured from the tail vein at 0, 30, 60, 90, and 120 min post-injection, following the same standardized protocol as OGTT. ITT results were normalized by blood glucose reduction percentage [(baseline glucose—time-point glucose)/baseline glucose × 100%] and analyzed via 120-min AUC to evaluate insulin sensitivity. If glucose dropped below 2.2 mmol/L, hypoglycemia was corrected with intraperitoneal 20% glucose (2 g/kg), and data after this time point were invalid.

### Echocardiography

#### Anesthetic induction and maintenance in experiment

To perform echocardiography, mice were anesthetized in a pre-flushed induction chamber with 3.0–3.5% isoflurane in oxygen (1.5 L/min) for 2–3 min until endpoints were met: loss of righting reflex, reduced spontaneous movement, and stabilized respiratory rate (60–80 breaths/min). Maintenance anesthesia was 1.0–1.5% isoflurane in oxygen (0.8–1.0 L/min) to balance immobility and physiological stability.

#### Echocardiographic measurement

Left ventricular function was evaluated by two-dimensionally guided M-mode echocardiography using a Vevo 2100 system (VisualSonics, Toronto, ON, Canada). Key parameters included left ventricular ejection fraction (LVEF), diastolic interventricular septal thickness (IVSTd), diastolic left ventricular posterior wall thickness (LVPWd), and diastolic left ventricular internal dimension (LVIDd).

#### Randomization and blinding of echocardiographic assessment

After random group allocation, animal IDs and group information were decoupled into anonymous identifiers. For echocardiography, animals were tested in a random order based on these identifiers (not reflecting group affiliation), and sonographers remained blinded to group assignments throughout assessments.

### Histology

#### Euthanasia protocol

Following the experiment, all mice were deeply anesthetized with 3.0–3.5% isoflurane in pre-flushed oxygen, followed by euthanasia via cervical dislocation. Biological death was verified by cessation of respiration (≥ 30 s), absent palpable heartbeat (≥ 1 min), and dilated, fixed pupils.

#### Sample preparation

To ensure diastolic cardiac arrest, each heart was injected with 10 mL of 10% potassium chloride, then rapidly transferred to ice-cold phosphate-buffered saline (PBS). Hearts were transversely trimmed into two segments: the upper segment was fixed in 4% paraformaldehyde at 4 °C for 24 h, then paraffin-embedded for hematoxylin and eosin (H&E) and Masson’s trichrome staining; the lower segment was snap-frozen in liquid nitrogen and stored at −80 °C for subsequent mRNA and protein extraction.

#### Histological staining and cardiac PRMT5 staining

Paraffin-embedded heart tissues were cut into 4–5 μm sections. H&E staining was used for histological evaluation, and Masson’s trichrome staining for collagen deposition; cardiomyocyte cross-sectional area and collagen deposition percentage were analyzed with a Zeiss Axio Vert.A1 Imager microscope (Carl Zeiss AG, Oberkochen, Germany).

For PRMT5 expression assessment, paraffin sections were processed for immunofluorescence: deparaffinization (xylene, gradient ethanol, distilled water), antigen retrieval (boiling 10 mM sodium citrate buffer, pH 6.0, microwave), permeabilization (0.1% Triton X-100) and blocking (5% BSA, 1 h at room temperature). Sections were incubated with PRMT5 primary antibody at 4 °C overnight, followed by Alexa Fluor 488-conjugated secondary antibody (1 h at room temperature, dark). Nuclei were stained with 1 μg/mL DAPI, images were captured via fluorescence microscope, and PRMT5 fluorescent intensity (relative to DAPI) was quantified using ImageJ software (Version 1.8.0).

#### Randomization and blinding of histological assessment

To eliminate group labeling, heart samples were assigned unique anonymous identifiers following removal of original group tags, then trimmed, fixed and embedded by a designated technician. Only anonymous identifiers were retained on paraffin blocks, and sections were randomized to avoid group inference by sectioning personnel. Furthermore, quantitative analysis of cardiomyocyte cross-sectional area, collagen deposition ratio and PRMT5 immunofluorescence intensity was conducted by two independent pathologists blinded to sample grouping using Image J software.

### Western blotting

Protein was extracted from cardiomyocytes or heart tissue using RIPA lysis buffer (Beyotime, Nantong, China). Equal amounts of protein were mixed with 5 × SDS loading buffer (Beyotime) and PBS to the same volume, then loaded onto 8%−15% SDS-PAGE gels for electrophoresis. Proteins were transferred to methanol-pretreated PVDF membranes (EMD Millipore) at 230 mA for 110 min, blocked with 5% non-fat milk for 1 h, and incubated with primary antibodies at 4 °C overnight. After washing, membranes were incubated with HRP-conjugated secondary antibodies at room temperature for 1 h. Blots were visualized with Tanon™ High-sig ECL Substrate (Tanon, Shanghai, China) using a ChemiDoc MP system (BIO-RAD, California, USA), and band intensities were analyzed via ImageJ software. Tubulin served as the internal control. Antibodies were shown in Table S1.

### Real-time quantitative polymerase chain reaction (Q-PCR)

To obtain total RNA, NRCMs and heart tissue were processed with Trizol Reagent (Invitrogen, USA), in accordance with the manufacturer's instructions. Then, approximately 2 μg total RNA was reverse-transcribed into first strand cDNA by using the reagents in RevertAid First Strand cDNA Synthesis Kit (Thermo Fisher Scientific, MA, USA). After that, the quantitative PCR (qPCR) of cDNA was conducted by using the SYBR Green qPCR Mix (TOYOBO) in a Roche LightCycler® 480 Real-Time PCR System (Roche). Expression quantification of mRNA were compared using the relative Ct method, where the amount of target normalized to the amount of endogenous control (β-actin) and relative to the control sample is given by 2^−∆∆Ct^. Primers used for qPCR analysis are listed in Table S2.

### Measurement of cell surface area and Immunofluorescence (IF) assay

#### Measurement of cell surface area

NRCMs in 24-well plates were fixed with 4% paraformaldehyde-PBS for 15 min, permeabilized with 0.3% Triton-100 for 10 min, then stained with 0.1% (v/v) rhodamine-phalloidin and DAPI. Images were captured via High Content Screening system (ArrayScanVTI, Thermo Fisher Scientific) and randomly renamed by a third party to mask group information. Cell surface area (CSA) was analyzed with Image J software from 6 random fields per group, and data were presented as fold change relative to the control group.

#### IF assay

Paraffin-embedded mouse myocardial tissues were cut into 4–5 μm-thick sections. The sections were deparaffinized in xylene three times for 5 min each, followed by gradual rehydration in a series of graded ethanol solutions (100%, 95%, 80% and 70%) for 2 min per concentration, and then rinsed with distilled water. Antigen retrieval was conducted in 10 mM sodium citrate buffer (pH 6.0) via microwave heating: high-power heating for 10 min, followed by natural cooling for another 10 min. Subsequently, the sections were permeabilized with 0.1% Triton X-100 diluted in PBS at room temperature for 10 min, and blocked with 5% bovine serum albumin (BSA) for 60 min to eliminate non-specific antibody binding. The sections were incubated overnight at 4 °C with primary antibodies targeting PRMT5 or Ndufs2 at a dilution of 1:200. After three consecutive PBS washes (5 min each), the samples were incubated with Alexa Fluor 488-conjugated anti-rabbit IgG (1:500) or Alexa Fluor 594-conjugated anti-mouse IgG (1:500) for 60 min at room temperature under dark conditions. Cell nuclei were counterstained using 1 μg/mL DAPI solution for 5 min. Finally, all stained sections were sealed with anti-fade mounting medium for subsequent observation.

Fluorescence imaging was performed using a Zeiss Axio Vert.A1 fluorescence microscope with identical parameters across all groups to minimize signal variability: excitation laser power 20%, exposure time 200 ms, detector gain 80 for Alexa Fluor 488; excitation laser power 25%, exposure time 300 ms, detector gain 90 for Alexa Fluor 594; excitation laser power 15%, exposure time 100 ms, detector gain 70 for DAPI. At least 6 random fields per group were captured. Fluorescence intensity was quantified using Image J software. PRMT5 and Ndufs2 signal intensities were normalized to corresponding DAPI intensity to correct for differences in section thickness and cell density. Data are presented as mean ± SD.

### Oxygen consumption rate analysis

NRCMs were isolated from mice, seeded into a Seahorse XF96 Culture Microplate (Agilent, Cat. No. 101085–004), and cultured overnight in XF Assay Medium (Seahorse Bioscience, Cat. No. 103680–100). Oxygen consumption rate (OCR) was measured with an Agilent Seahorse Xfe96 Extracellular Flux Analyzer (hydrated probes) per manufacturer’s instructions, at baseline and after sequential injection of oligomycin (1 μM, MCE, Cat. No. HY-N6782), FCCP (1 μM, MCE, Cat. No. HY-100410), and antimycin A (0.5 μM, Sigma-Aldrich, Cat. No. A8674). After OCR measurement, total protein was extracted (CST Cell Lysis Buffer, Cat. No. 9803), and concentration was quantified via BCA kit (ThermoFisher, Cat. No. 23225). OCR values were normalized to protein concentration for standardization.

### Small interference RNA and plasmid transfection

PRMT5-targeting small interfering RNA (si-PRMT5) and negative control siRNA (si-NC) were purchased from RibBio (Guangzhou, China). For transfection, NRCMs at 60% confluency in 6-well plates were cultured in Opti-MEM® I Medium (Gibco) with a mixture of 5 μl Lipofectamine 2000 (Invitrogen) and 40 nmol siRNA. Plasmids (wild-type PRMT5, Flag-tagged wild-type Ndufs2, Flag-tagged mutant Ndufs2 [R118H]) were constructed in pcDNA3.1(+) vector by VIC GENE (Guangzhou, China). Flag-tagged Ndufs2 mutant (K108R/K112R/K127R) was generated via site-directed mutagenesis (QuikChange II Kit, Agilent) of lysine residues 108, 122, and 127 to arginine. NRCMs in 6-well plates were transiently transfected with 2 μg plasmids using the same method as siRNA transfection; medium was replaced with fresh 10% FBS-DMEM after 6 h, and cells were cultured for 48 h before harvesting. The sequences of siRNAs and plasmid are listed in Supplementary Table S3.

### Co-immunoprecipitation (Co-IP)

Cells or tissue were lysed by IP lysis buffer (Nantong, Jiangsu, China). Briefly, 500 μg protein lysate was used for immunoprecipitation, the protein lysate was separately incubated with targeted primary antibody overnight or anti-mouse IgG antibody (as control) at 4℃ overnight. After that, the protein lysate-primary antibody mixture was incubated with 30 μl protein A/G beads (MedChemExpress, USA) at 4℃ for 2 h. The beads were collected by magnetic rack and were boiled with 30 μl of 2 × loading buffer, the immunoprecipitated proteins were assayed by Western blotting, as described above.

### Liquid chromatography-tandem mass spectrometry (LC–MS/MS) analysis

#### Sample preparation and loading

Protein was extracted from heart tissue using UA lysis buffer (8 M urea, 100 mM Tris/HCl, pH 8.5) and quantified by Bradford assay. Approximately 20 μg of eluted proteins from co-IP were separated by 12% SDS-PAGE at 14 mA for 90 min and visualized with Coomassie blue. Target bands were excised, decolorized, dehydrated, reduced with DTT, alkylated with IAA, and digested with sequencing-grade trypsin for 15–18 h. The reaction was terminated by adding TFA to a final concentration of 0.1% (pH ≤ 3), and peptides were desalted using C18 SPE cartridges and freeze-dried. Methylated peptides were enriched using pre-treated Anti-Symmetric-Di-Methyl Arginine Motif immunoaffinity beads in pre-cooled IAP buffer at 4 ℃ for 1.5 h. Beads were washed sequentially with IAP buffer and water, followed by elution twice with 0.15% TFA at room temperature; eluates were desalted with C18 STAGE Tips. LC–MS/MS analysis was performed using a NanoElute HPLC system coupled with a timsTOF Pro mass spectrometer in positive ion mode. Peptides were separated on an AUR2-25075C18A-CSI column (25 cm × 75 μm ID, 1.6 μm C18) at 300 nL/min using 0.1% formic acid in water (buffer A) and 0.1% formic acid in acetonitrile (buffer B). Data were acquired in PASEF mode with a 1.5 kV ion source voltage, 100–1700 m/z scan range, 1.16 s cycle window, and 0.4 min dynamic exclusion.

#### Threshold criteria and data normalization

Raw MS data were processed with PEAKS Studio (Bioinformatics Solutions Inc.) and searched against the UniProt mouse database (release 2023). Search parameters included carbamidomethylation (C, fixed), oxidation (M, variable), and symmetric di-methylation (R, variable). The false discovery rate (FDR) was controlled at 1%. For differential analysis between EPZ-treated and control groups, peptides with fold change (FC) ≥ 1.5 or ≤ 0.67 and p < 0.05 were considered significantly altered.

Missing intensity values were imputed using the k‑nearest neighbors (k‑NN) algorithm. Peptides intensities were median‑normalized and log2‑transformed before statistical testing.

#### Target validation

Candidate substrates were prioritized using three criteria: (1) involvement in diabetic cardiomyopathy or oxidative phosphorylation KEGG pathways; (2) peptide coverage ≥ 2; (3) spectral count ≥ 3. The top candidate Ndufs2, was validated via orthogonal approaches: (1) independent Co‑IP and western blotting confirming PRMT5 interaction and SDMA modification; (2) manual MS/MS spectrum annotation verifying the 28.03 Da mass shift corresponding to R118 symmetric dimethylation; (3) site‑directed mutagenesis (R118H) abolishing PRMT5‑mediated SDMA.

### Recombinant protein purification

GST‑tagged wild‑type (WT) and catalytic mutant (R367A/R368A) PRMT5 were cloned into pGEX‑6P‑1, and Flag‑tagged WT and R118H mutant Ndufs2 were inserted into pET‑28a. All plasmids were confirmed by Sanger sequencing and transformed into Escherichia coli BL21 (DE3). Bacteria were grown in LB medium at 37 °C to OD₆₀₀ = 0.6, then induced with 0.5 mM IPTG at 16 °C for 16 h.

For GST‑PRMT5 purification, cell pellets were sonicated in GST lysis buffer (50 mM Tris‑HCl pH 7.5, 150 mM NaCl, 1 mM DTT, 1% Triton X‑100, 1 mM PMSF). After centrifugation at 12,000 g for 30 min at 4 °C, supernatants were incubated with glutathione‑Sepharose 4B beads. Beads were washed five times and eluted with buffer containing 10 mM reduced glutathione. For Flag‑Ndufs2 purification, cells were lysed in Flag lysis buffer (50 mM Tris‑HCl pH 8.0, 200 mM NaCl, 10 mM imidazole, 1 mM DTT, 1% Triton X‑100, 1 mM PMSF). Supernatants were incubated with Ni‑NTA agarose beads, washed five times with 50 mM imidazole, and eluted with 250 mM imidazole. Purified proteins were concentrated with Amicon Ultra‑15 filters, quantified by BCA assay, and validated for purity (> 90%) by 12% SDS‑PAGE and Coomassie brilliant blue staining.

### GST pull-down assay

For the in vitro binding assay, 2 μg of GST‑PRMT5 (wild‑type or mutant) or GST alone (negative control) was immobilized on 20 μL glutathione‑Sepharose 4B beads in binding buffer (50 mM Tris‑HCl pH 7.5, 150 mM NaCl, 1 mM DTT, 0.5% NP‑40, 5% glycerol) at 4 ℃ for 1 h. After immobilization, 4 μg of purified Flag‑Ndufs2 (wild‑type or R118H mutant) was added, and the mixture was incubated at 4 ℃ for 2 h with gentle rotation. Beads were washed five times with binding buffer to remove unbound proteins, and bound proteins were eluted by boiling in 2 × SDS loading buffer for 5 min. Eluted proteins were analyzed by Western blotting using anti‑GST and anti‑Flag antibodies (both 1:5000). As this was a purified protein system, tubulin was not used as an internal control; protein loading was normalized by input.

### *Cell*-free methylation assay

For the in vitro methylation assay, 2 μg of GST‑PRMT5 (wild‑type or mutant), 4 μg of Flag‑Ndufs2 (wild‑type or R118H mutant), and 5 μM SAM (Sigma‑Aldrich) were mixed in methylation buffer (50 mM Tris‑HCl pH 8.0, 10 mM MgCl₂, 1 mM DTT) to a final volume of 50 μL. Reactions were incubated at 37 ℃ for 3 h, terminated by the addition of 2 × SDS loading buffer and boiling for 5 min, and subsequently analyzed by Western blotting using anti‑SDMA and anti‑Flag antibodies (both 1:5000).

### Clinical research on serum PRMT5

#### Statement for ethics compliance and informed consent for clinical retrospective study

This retrospective study investigating serum PRMT5 levels in patients with and without DCM was conducted in accordance with the Declaration of Helsinki and relevant ethical guidelines for human research. Written informed consent was obtained from all participants before enrollment and blood collection, with clear explanation of the study purpose, sample usage, storage procedures, and de-identification protocols to ensure privacy protection. No samples or clinical data were used without explicit written consent. The study protocol was approved by the Institutional Review Board of the University of Hong Kong (Approval No.: [2022]246).

#### Criteria of study subjects and collection of baseline characteristics data

This retrospective study was conducted using medical data from the territory-wide Clinical Data Analysis Reporting System (CDARS, initiated by Hong Kong’s Hospital Authority, which provides over 80% of local inpatient services) and serum samples from eligible patients. CDARS has prospectively collected demographic data, diagnoses, medications, interventions, laboratory results, and hospital visit records since 1993. A total of 97 participants who met the inclusion criteria and were recorded in the cardiology department medical database of Queen Mary Hospital, The University of Hong Kong, from January 2020 to June 2025 were enrolled. All personal information was de-identified in CDARS to ensure privacy. Dilated cardiomyopathy (DCM) was defined by International Classification of Diseases, Eleventh Revision (ICD-11) code BC43.7. The study population included non-DCM and DCM participants: non-DCM participants had FBG ≤ 6.1 mmol/L, HbA1c ≤ 6.5%, no congenital heart defects, and no coronary artery disease, valvar disease, or conventional cardiovascular risk factors (hypertension, diabetes, dyslipidemia). DCM participants had hyperglycemia (FBG > 6.1 mmol/L or HbA1c ≥ 6.5%), no overt coronary artery disease, valvar disease, or conventional cardiovascular risk factors, and cardiac structural/functional abnormalities (myocardial fibrosis, remodeling, diastolic/systolic dysfunction, heart failure) on echocardiography and cardiac magnetic resonance. Clinical characteristics extracted from medical records included age, sex, NT-proBNP, BMI, HbA1c, and echocardiographic parameters (IVSTd, LVPWd, LVIDd, LVEF, LVMi, E/e’).

#### Serum sample preparation and follow-up analysis

Serum samples were extracted from blood sample reserved in the Blood Sample Biorepository of the Department of Cardiology, the University of Hong Kong, Queen Mary Hospital. To obtain serum samples, the blood was centrifuged at 1500 g for 15 min, followed by centrifugation at 13,000 g for two minutes. Serum PRMT5 level was measured according to the manufacturer’s instructions in PRMT5 ELISA Kit (LMAI Biotechnology Corp, China).

The follow-up stage involved monitoring of hospital readmission rates. Commencing from participant recruitment and initial serum sample collection, the follow-up extended over a median duration of 30.3 months.

### Statistical analysis

All statistical analyses were performed following verification of data normality using the Shapiro–Wilk test. Data are presented as mean ± standard deviation (SD), with Cohen’s d value and its 95% confidential interval (95% CI) for animal and cellular experimental results, a two-sided P < 0.05 was considered to indicate statistical significance.

#### Sample size justification

Sample size calculation and power analysis were performed a priori using GPower 3.1 software and standard statistical formulae to ensure adequate statistical power for all in vivo and in vitro experiments. For two-group comparisons (Student’s t-test) in animal and cellular experiments, sample size was calculated as: $n=\frac{{\left({z}_{1-\alpha /2}+{z}_{1-\beta }\right)}^{2}\times 2{\sigma }^{2}}{{\delta }^{2}}$, where type I error rate α = 0.05, statistical power (1-β) = 0.80, critical Z-value for a two-sided test Z _1−α/2_ = 1.96, critical Z-value for 80% power Z _1−β/2_ = 0.84, indicator of standard deviation versus indicator demonstrates minimum biologically meaningful difference between groups σ/δ = 0.40. Based on the calculation above, the minimum required sample size was *n* = 2.5088 per group. Therefore, *n* = 6 each group is suitable for two-group comparisons. For multi-group comparisons in animal and cellular experiments, sample size estimation for one-way ANOVA was conducted based on the F-distribution and noncentrality parameter ϕ = f $\sqrt{nk}$, where f is Cohen’s effect size, k is the number of groups, and n is the sample size per group. The type I error rate was set at α = 0.05, statistical power at 1-β = 0.80, with k = 4 experimental groups and an assumed Cohen’s f = 1.00. According to the noncentral F-test criterion, at least 5 replicates per group were required to achieve the preset statistical power. Therefore, n = 6 each group is suitable for multi-group comparisons.

For calculation of clinical sample size, sample size was calculated using the Hanley–McNeil formula for area under curve (AUC) of receiver operating characteristic $\left(ROC\right):{N}_{total}=\frac{{\left(1+{\kappa }^{2}\right)*\left({Z}_{1-\alpha /2}+{Z}_{1-\beta }\right)}^{2}}{{{\kappa }^{*}\left({\Phi }^{-1}\left(AUC\right)\right)}^{2}}$, where ${AUC}_{1}=0.65$ (hypothesized diagnostic AUC of serum PRMT5), AUC_0_ = 0.5(null hypothesis), type I error rate α = 0.05, statistical power (1-β) = 0.80, critical Z-value for a two-sided test Z_1−α/2_ = 1.96, critical Z-value for 80% power Z_1−β/2_ = 0.84, *κ* = N_non-DCM_/N_DCM_ = 58/39 = 1.487 (allocation ratio), ${\Phi }^{-1}$ = inverse normal distribution function. The calculation yielded a minimum required total sample size of 84 participants (50 for non-DCM and 34 for DCM). A total of 97 participants were recruited in this study, consisting of 58 non-DCM and 39 DCM cases. The sample size was sufficient to meet the standard statistical requirements.

#### Power calculation

Power analysis were performed using GPower 3.1 software for all in vivo and in vitro experiments. For two-sample t-tests, the power $1-\beta =\Phi \left(d\sqrt{\frac{n}{2}} -{Z}_{1-\alpha /2}\right)$, with *n* = 6 indicates sample size per group, d indicates effect size of Cohen’s d, Z_1−α/2_ = 1.96 indicates Z-value for a two-sided test. Φ denotes the cumulative distribution function of the standard normal distribution, which can be obtained from standard normal tables, statistical software like GPower 3.1. For one-way ANOVA analysis, the power 1-β = 1 − F _crit_ (f ^2^n, k − 1, N − k), with *n* = 6 indicates sample size per group, k = 4 indicates the number of experimental groups, *N* = 24 indicates total sample size, f indicates Cohen’s f (effect size for ANOVA), F_crit_ indicates the critical value of the F-distribution. The values of F_crit_ can be obtained from F-tables or statistical software GPower 3.1.

For clinical ROC analysis, the power 1-β was calculated using the formula: $1-\beta =\Phi \left(\sqrt{\frac{N\times \kappa }{1+\kappa }}\times {\Phi }^{-1}\left({AUC}_{1}\right)\right)-{Z}_{1-\alpha /2}$, within *N* = 97 indicates total sample size, κ indicates the ratio of non-DCM to DCM participants, AUC indicates the expected area under the ROC curve, Z_1-α/2_ = 1.96 indicates the Z-value for a two-sided test. Φ denotes the cumulative distribution function of the standard normal distribution, Φ^−1^ denotes the inverse cumulative distribution function, both of which can be obtained from standard normal tables or statistical software like MedCalc.

#### Statistical analysis in animal and cellular experiments

Comparisons between two independent groups were performed using unpaired two-tailed Student’s t-tests. Multiple group comparisons were analyzed by one-way analysis of variance (ANOVA) followed by Tukey’s post-hoc test with Bonferroni correction. All analyses were conducted using GraphPad Prism 5.01 (GraphPad Software, Inc., La Jolla, CA, USA).

#### Statistical analysis in clinical data

For clinical research data, statistical analyses were performed using IBM SPSS Statistics Version 20 (IBM Corp., Armonk, NY, USA) as follows: (1) Correlation analysis: Pearson’s correlation analysis assessed linear correlations between serum PRMT5 levels and serum lactate levels or lactate/pyruvate (L/P) ratios, with *P*-values determined by the Pearson’s correlation test. (2) Diagnostic value assessment: ROC analysis evaluated the diagnostic efficacy of serum PRMT5 for differentiating DCM patients from non-DCM participants, the AUC was calculated to quantify discriminatory ability, with *P*-values derived from ROC curve analysis. (3) Independent association analysis: Multivariable logistic regression analysis identified independent associations between serum PRMT5 levels and DCM, adjusted for potential confounders (age and sex). *P*-values were obtained via the Wald test, and results were visualized as forest plots. (4) Categorical data analysis: Pearson’s χ^2^ test was used for comparisons of categorical variables, with *P*-values determined by the test. (5) Survival analysis: Kaplan–Meier survival curves were plotted to assess the risk of hospital readmission, with *P*-values determined via the log-rank test. All clinical correlation and regression analyses were corrected using Benjamini–Hochberg false discovery rate (FDR) for multiple endpoints.

#### Statistical analysis in IP-MS proteomics data

Multiple comparison correction was applied to control type I error. For high‑IP‑MS proteomics datasets, the Benjamini–Hochberg FDR correction was used. Peptides with fold‑change ≥ 1.5 or ≤ 0.67 and an unadjusted *P* < 0.05 were considered differentially modified. Only those with FDR < 0.05 were retained as statistically significant. For KEGG pathway enrichment analysis, enrichment *p*‑values were adjusted by FDR, and pathways with FDR < 0.05 were considered significant. For small-scale pre-specified comparisons in animal and cellular experiments, the Bonferroni correction was used.

### Reagents and drugs

Other materials like inhibitors utilized in this study were listed in supplementary document Table S4-S5.

## Results

### PRMT5 is downregulated in heart of murine DCM model

To investigate cardiac PRMT5 expression in DCM, we established a murine DCM model via 24-week high-fat diet (HFD) feeding combined with a single intraperitoneal injection of streptozotocin (STZ, 35 mg/kg; Fig. 1a). HFD/STZ-treated mice exhibited significant cardiac abnormalities compared to controls, including cardiac enlargement, increased heart weight, and elevated heart weight-to-tibia length ratio (Fig. 1b-d). Histological analysis revealed disrupted myocardial architecture, enhanced collagen deposition, and augmented cardiomyocyte cross-sectional area, indicating phenotypes of pathological cardiac remodeling in HFD/STZ-treated mice (Fig. 1e–h).

Metabolically, DCM mice displayed typical diabetic phenotypes with impaired glucose tolerance and insulin resistance, as reflected by an increased AUC in the OGTT and ITT and higher FBG level (Fig. 1i–k). Echocardiographic analysis demonstrated left ventricular structural remodeling and functional impairment, characterized by increased IVSTd and LVPWd, a trend toward reduced LVIDd, and decreased LVEF (Fig. 1l-p). Consistent with functional deficits, myocardial ATP levels were markedly depleted in DCM mice (Fig. 1q).

Molecular analyses revealed that PRMT5 was significantly downregulated at protein, immunofluorescence, and mRNA levels in DCM hearts, while hypertrophy markers atrial natriuretic factor (ANF) and β-myosin heavy chain (β-MHC) were markedly upregulated (Fig. 1r–t).

Collectively, these data suggest a potential association between PRMT5 deficiency and pathological cardiac remodeling in DCM.

### PRMT5 exerts cardioprotective effects in DCM models

To define PRMT5's functional role in DCM, we performed in vivo gain-of-function experiments via intramyocardial injection of PRMT5-overexpressing adenovirus (Ad-PRMT5) or empty vector (Ad-vector) at Week 12 of DCM induction (Fig. 2a). Mice were divided into four groups: Ad-vector + CON (Group 1), Ad-vector + HFD/STZ (Group 2), Ad-PRMT5 + CON (Group 3), and Ad-PRMT5 + HFD/STZ (Group 4). Pathological analyses showed that Ad-vector + HFD/STZ mice exhibited exacerbated cardiac abnormalities compared with Ad-vector + CON, including increased collagen deposition, CSA, HW, and HW/TL ratio (Fig. 2b-d, 2f-i). Echocardiographic results presented impaired cardiac function in Ad-vector + HFD/STZ mice, as evidenced by decreased LVEF, along with structural remodeling characterized by increased IVSTd and LVPWd (Fig. 2e, 2j-m). At the molecular level, Ad-vector + HFD/STZ mice showed upregulated ANF and β-MHC, and reduced cardiac ATP levels (Fig. 2n-o). Notably, Ad-PRMT5 treatment significantly attenuated these DCM-related phenotypes: compared with Ad-vector + HFD/STZ, Ad-PRMT5 + HFD/STZ mice showed reduced collagen deposition, CSA, HW, and HW/TL ratio, as well as improved LVEF and cardiac ATP levels (Fig. 2f–p).

**Fig. 3:**
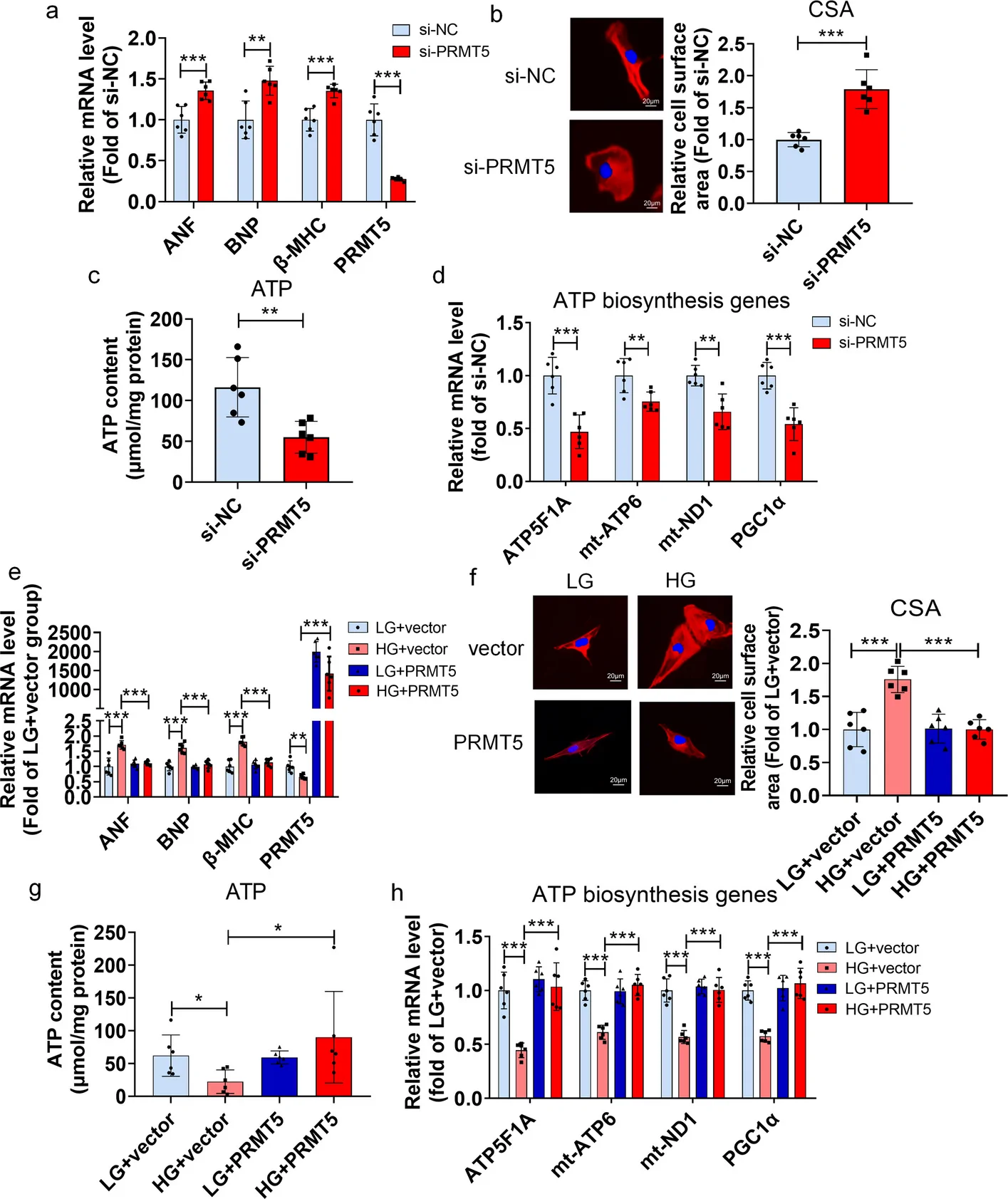
PRMT5 protects against DCM in vitro. **a** mRNA levels of hypertrophic markers (ANF, BNP, β-MHC) and PRMT5 in NRCMs transfected with si-NC or si-PRMT5. **b** Cardiomyocyte CSA in si-NC and si-PRMT5 groups. Scale bar = 20 μm. **c** Intracellular ATP levels in si-NC and si-PRMT5 groups. **d** mRNA levels of ATP biosynthesis-related genes (ATP5F1A, mt-ATP6, mt-ND1, PGC1α) in si-NC and si-PRMT5 groups. **e**–**h** PRMT5 overexpression effects on hypertrophic markers and PRMT5 mRNA (**e**), CSA (f; scale bar = 20 μm), ATP levels (**g**), and ATP biosynthesis-related gene expression (**h**) under low glucose plus vector (LG + vector), high glucose plus vector (HG + vector), low glucose plus PRMT5 overexpression (LG + PRMT5) and HG plus PRMT5 overexpression (HG + PRMT5) conditions. Data are presented as mean ± SD. *P* values by unpaired two-tailed Student's t-test (**a**–**d**) or one-way ANOVA with Tukey's post-hoc test (**e**–**h**), *n* = 6

To further determine the function of PRMT5 in cardiomyopathy, we performed loss-of-function and gain-of-function experiments by transfecting NRCMs with either PRMT5-targeting siRNA (si-PRMT5) or PRMT5 overexpression plasmids, with transfection efficiency verified by Western blotting (Fig. S1e-f). PRMT5 knockdown via si-PRMT5 upregulated hypertrophy markers (ANF, BNP, and β-MHC) and increased CSA (Fig. 3a–b). Metabolically, si-PRMT5 reduced ATP levels and suppressed expression of ATP synthesis-related genes, including ATP5F1A, mt-ATP6, mt-ND1, and PGC1α (Fig. 3c-d). In contrast, PRMT5 overexpression alleviated hyperglycemia-induced pathological changes in NRCMs. Compared with HG treatment with vector transfection (HG + vector), HG + PRMT5 normalized hypertrophy markers (ANF, BNP, and β-MHC), reduced augmented CSA, increased ATP production, and restored the expression of ATP biosynthetic genes (ATP5F1A, mt-ATP6, mt-ND1, and PGC1α) (Fig. 3e-h). Taken together, these in vitro and in vivo findings support that PRMT5 exerts cardioprotective effects in DCM models.

### The SDMA activity of PRMT5 regulates OXPHOS and ATP biogenesis in cardiomyocytes

**Fig. 4:**
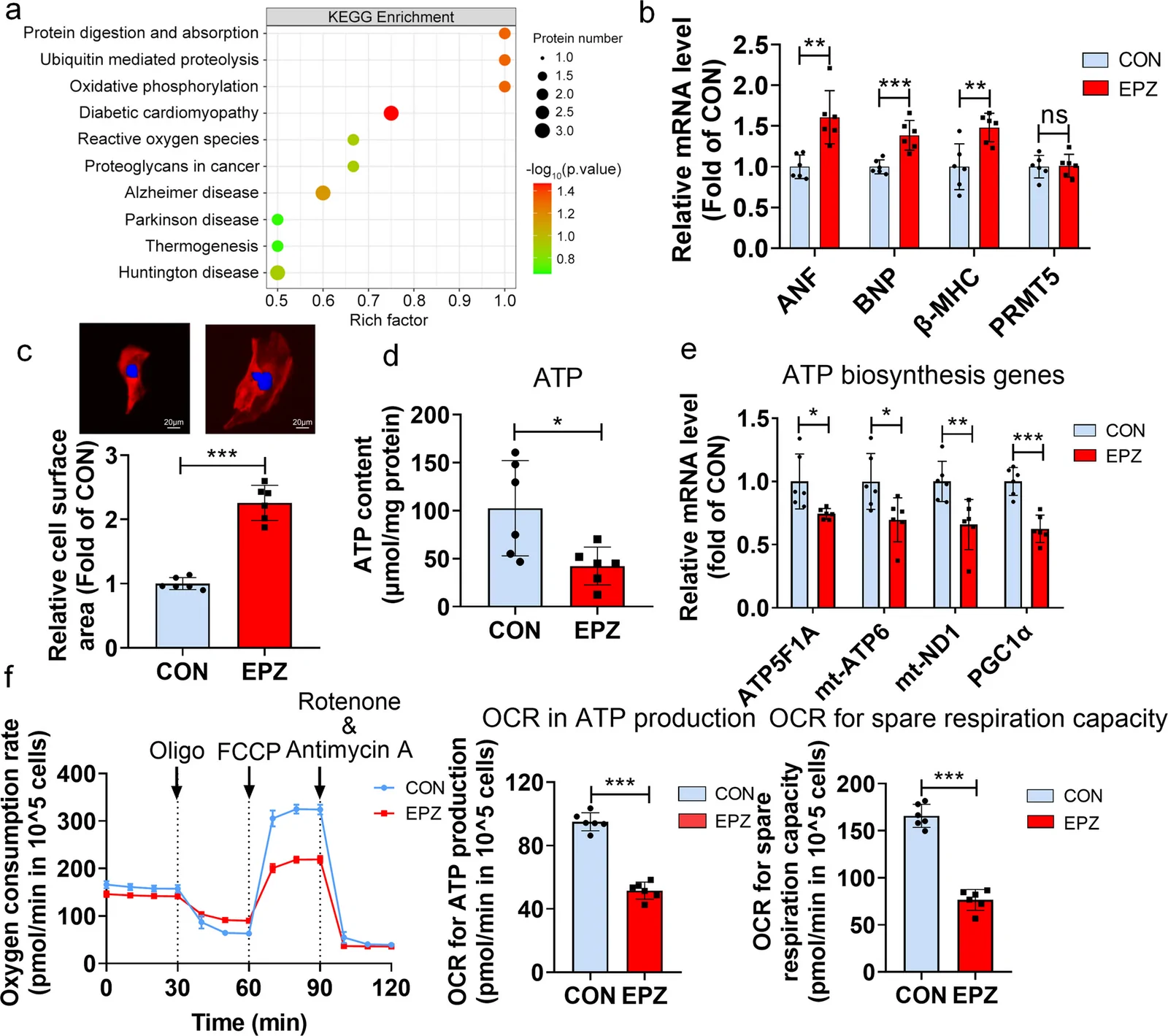
SDMA mediated by PRMT5 promotes OXPHOS and ATP biosynthesis in cardiomyocytes. **a** KEGG analysis of potential processes regulated by the SDMA activity of PRMT5 in NRCMs. **b** qPCR analysis of hypertrophy-related genes (ANF, BNP, β-MHC) and PRMT5 in NRCMs with or without the PRMT5 enzymatic inhibitor EPZ. **c** Cardiomyocyte CSA in si-PRMT5 and si-NC groups. Scale bar = 20 μm. **d** Intracellular ATP levels in NRCMs with or without EPZ treatment. **e** qPCR analysis of ATP biosynthesis-related genes (ATP5F1A, mt-ATP6, mt-ND1, PGC1α) in NRCMs with or without EPZ treatment. **f** Oxygen consumption rate (OCR) in cardiomyocytes with or without EPZ treatment is shown in the left panel. OCR in ATP production and OCR for spare respiratory capacity are calculated and shown in the middle and right panel. Data are presented as mean ± SD. *P* values by unpaired two-tailed Student's t-test, *P < 0.05; **P < 0.01; ***P < 0.001, ns, no significance, *n* = 6

The arginine methyltransferase PRMT5 is known to regulate cardiac homeostasis, in part through its symmetric dimethylarginine (SDMA) activity on substrate proteins. To investigate the enzymatic mechanism underlying PRMT5's cardioprotective effects in DCM, we treated NRCMs with EPZ015666 (EPZ), a selective SDMA inhibitor (validation in Fig. S1g). To rule out potential off-target effects, we examined the expression of other PRMT family members and global asymmetric dimethylation (ADMA) levels after EPZ treatment. No significant changes were observed (Supplementary Fig. S2b-c), suggesting the specificity of EPZ under these experimental conditions. KEGG pathway enrichment analysis of IP-MS data identified the top 10 biological processes regulated by PRMT5, including ubiquitin-mediated proteolysis (Rank 2), oxidative phosphorylation (Rank 3), and diabetic cardiomyopathy (Rank 4) (Fig. 4a), which were consistent with DCM's core pathological mechanisms. Functional analyses showed that EPZ treatment recapitulated PRMT5 knockdown phenotypes without influencing PRMT5 levels, including upregulated hypertrophy markers (ANF, BNP, and β-MHC, Fig. 4b), increased CSA (Fig. 4c), reduced ATP levels (Fig. 4d), and downregulated ATP biosynthesis genes (ATP5F1A, mt-ATP6, mt-ND1, and PGC1α, Fig. 4e). These results supported that the effects of EPZ are likely mediated by PRMT5 inhibition rather than off-target effects (Fig. 3a-d). Oxygen consumption rate (OCR) measurements further demonstrated PRMT5's role in OXPHOS regulation: EPZ treatment reduced total OCR and specifically suppressed ATP production-linked OCR and spare respiratory capacity (Fig. 4f), which are key indicators of ETC Complex I/III/IV function. These data demonstrate that PRMT5's SDMA activity plays an important role in maintaining mitochondrial bioenergetics and suppressing cardiomyocyte hypertrophy.

### PRMT5 maintains ETC Complex I activity via SDMA modification of Ndufs2 at R118 residue

**Fig. 5:**
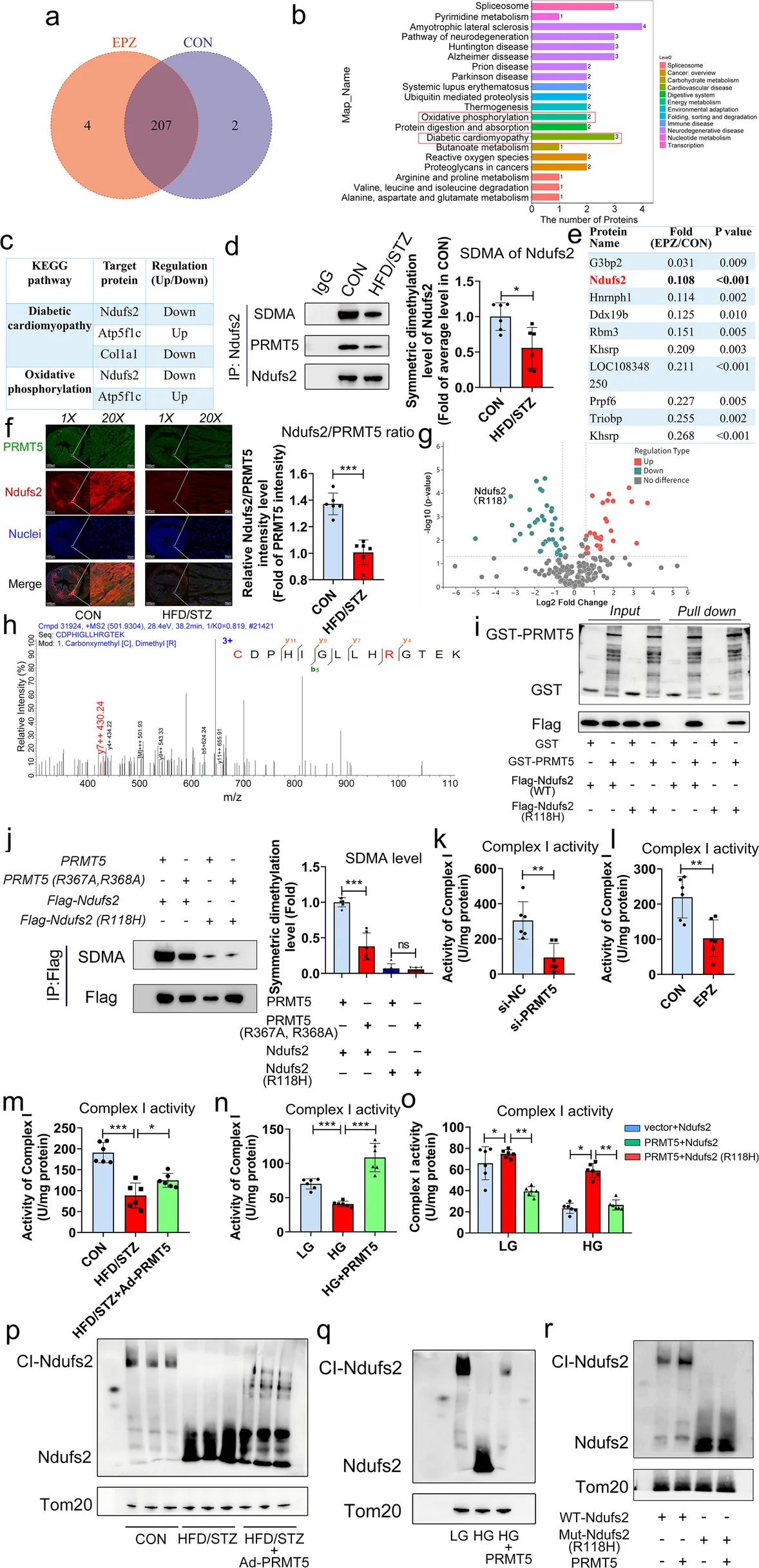
PRMT5 enhances Complex I activity via SDMA of Ndufs2 at R118. **a** Peptide Venn diagram showing number of whole-proteome proteins regulated by PRMT5. **b** Distribution of proteins with significantly differential SDMA modification levels across KEGG pathways (EPZ vs CON). **c** Proteins with significantly altered SDMA levels in the diabetic cardiomyopathy and oxidative phosphorylation pathways. **d** Top ten proteins with the most pronounced reduction in SDMA levels. **e** Left pannel: immunofluorescence staining of Ndufs2 (red) and PRMT5 (green) in heart sections from CON and HFD/STZ mice. DAPI (blue). Right pannel: quantification of relative Ndufs2/PRMT5 fluorescence intensity ratio. Scale bars = 1000 μm (left) and 50 μm (right). **f** Co-IP assay showing Ndufs2 SDMA levels in heart tissues in CON vs HFD/STZ. **g** IP-MS analysis (NRCMs, EPZ vs CON) identifying target proteins and modified arginine residues (fold change ≥ 1.5, *P* < 0.05; grey, unchanged; red, upregulated; green, downregulated). (h) Mass spectrometry identification of the Ndufs2 peptide and arginine residue modified by PRMT5. **i** GST pull-down assays showing direct binding between PRMT5 and WT- or R118H-mutant Ndufs2. **j** Co-IP assays showing SDMA levels of WT- or R118H-mutant Ndufs2 in NRCMs expressing WT-PRMT5 or catalytic-mutant PRMT5 (R367A/R368A). (**k**–**n**) Complex I activity measured by ELISA in NRCMs transfected with si-NC or si-PRMT5 (k), treated with or without EPZ (l), in heart tissues of CON, HFD/STZ, and HFD/STZ + Ad-PRMT5 groups (**m**), and in NRCMs under LG, HG, and HG + PRMT5 conditions (**n**). (**o**) Complex I activity in NRCMs transfected with WT-Ndufs2 or R118H-mutant Ndufs2, with or without PRMT5, under LG and HG conditions. (p–r) BN-PAGE analysis of Complex I integrity in heart tissues of CON, HFD/STZ, and HFD/STZ + Ad-PRMT5 groups (p), in NRCMs under LG, HG, and HG + PRMT5 conditions (**q**), and in NRCMs transfected with WT- or R118H-mutant Ndufs2 with or without PRMT5 (**r**). Tom20 was used as mitochondrial reference. Data are presented as mean ± SD. *P* values by unpaired two-tailed Student's t-test (**e**, **f**, **k** and **l**) or one-way ANOVA (**j**, **m**, **n** and **o**), *P < 0.05; **P < 0.01; ***P < 0.001, ns, no significance, *n* = 6

To identify PRMT5 substrates in DCM, we performed IP-MS in CON and EPZ-treated NRCMs and identified 207 proteins with differential SDMA modification (Fig. 5a). KEGG pathway analysis highlighted two DCM-related pathways (diabetic cardiomyopathy and oxidative phosphorylation), with Ndufs2 (NADH dehydrogenase [ubiquinone] iron-sulfur protein 2) exhibiting the most significant reduction in SDMA level and ranking second among all candidates (Fig. 5b-d). Immunofluorescence and co-immunoprecipitation (Co-IP) indicated reduced PRMT5-Ndufs2 interaction and decreased Ndufs2 SDMA level in DCM hearts (Fig. 5e-f). Furthermore, volcano plot indicated that R118 of Ndufs2 is the residue with differential SDMA modification (Fig. 5g). Among all detected SDMA peptides, the peptide containing R118 of Ndufs2 showed the most significant reduction in SDMA level upon PRMT5 inhibition (fold change = 0.108, *P* < 0.001, rank 2 in the global SDMA downregulated list, Fig. 5d, Supplementary List-Peptides with significant reduction in SDMA level). To examine this result, we performed detailed MS/MS spectrum annotation of the target peptide "CDPHIGLLHRGTEK" containing R118 residue of Ndufs2. Key y-ion (y7 + +) containing R118 (860.48 Da) showed a mass shift of 28.03 Da compared to the unmodified peptide (832.45 Da), confirming SDMA modification on R118 residue of Ndufs2 (Fig. 5h).

To test the direct interaction and catalytic modification between PRMT5 and Ndufs2, we performed GST pull-down and cell-free methylation assays using purified recombinant proteins. GST pull-down results showed that GST-PRMT5 (WT) efficiently pulled down Flag-Ndufs2 (WT), while weak binding was detected between GST-PRMT5 (WT) and Flag-Ndufs2 (R118H) (Fig. 5i, Lane 6 & 8). Notably, the catalytically inactive PRMT5 mutant (R367A/R368A), serving as a genetic inhibition control, still bound to Flag-Ndufs2 (WT), indicating that the interaction is independent of PRMT5's methyltransferase activity (Supplementary Fig. S2a, Lane 2 & 3). GST tag alone did not bind to either Ndufs2 WT or R118H, ruling out non-specific binding (Fig. 5i, Lane 5 & 7). Cell-free methylation assays further demonstrated that GST-PRMT5 (WT) enhanced the SDMA signal of Flag-Ndufs2 (WT) in the presence of SAM. In contrast, no obvious change in SDMA level was observed in reactions containing GST-PRMT5 (catalytic mutant) or Flag-Ndufs2 (R118H) (Supplementary Fig. S2a). Similarly, Co-IP validation via mutant Ndufs2 (R118H) in NRCMs showed that PRMT5 only increased SDMA levels in wild-type (WT)-Ndufs2 rather than R118H-mutant Ndufs2 (Fig. 5j), further demonstrating that R118 is the critical SDMA site targeted by PRMT5.

To rule out the possibility of compensatory upregulation of other PRMTs upon PRMT5 inhibition, we assessed the expression and activity of Type I PRMTs, a series of enzymes responsible for asymmetric di-methylation (ADMA), including PRMT1, PRMT2, PRMT3, PRMT4, PRMT6 and PRMT8 in EPZ-treated cardiomyocytes. Additionally, other PRMTs including PRMT7 and PRMT9 were also examined in EPZ-treated cardiomyocytes. As a result, no significant changes were observed in the protein levels of these PRMTs or in global ADMA marks (Supplementary Fig. S2b-c), indicating that PRMT5 inhibition does not elicit a compensatory response from other PRMT family members. Consistent with this, knockdown of PRMT5 by si-PRMT5 also failed to alter global ADMA levels (Supplementary Fig. S2d). These results indicate that PRMT5 is a key enzyme mediating SDMA modification of Ndufs2 at R118 in the context of DCM, and its loss cannot be functionally compensated by other PRMTs under these conditions.

Ndufs2 is the catalytic core of ETC Complex I. To investigate the functional importance of PRMT5-mediated SDMA modification of Ndufs2 for Complex I, we explored the activity and integrity of Complex I. Functional assays showed that both EPZ treatment and PRMT5 knockdown (si-PRMT5) decreased Complex I activity, while EPZ treatment alone did not affect the activities of Complexes II, III, and IV (Fig. 5k-l, Supplementary Fig. S2e-f). DCM hearts also exhibited reduced Complex I activity, which was restored by PRMT5 overexpression via Ad-PRMT5 (Fig. 5m). Similarly, PRMT5 overexpression rescued high glucose-induced Complex I impairment, while no significant changes were observed in the activities of Complexes II, III, and IV under the same conditions (Fig. 5n, Supplementary Fig. S2g).

Furthermore, functional experiments in NRCMs support the critical role of R118. PRMT5 overexpression together with WT-Ndufs2 moderately increased Complex I activity and ATP level under basal (LG) conditions and markedly rescued high glucose-induced Complex I impairment, whereas such rescue was not observed in NRCMs expressing the R118H mutant (Fig. 5o, Supplementary Fig. S2h). Additionally, we also assessed Complex I integrity. In DCM mice, high-molecular-weight intact Complex I bands of Ndufs2 were decreased and low-molecular-weight unassembled Ndufs2 bands increased. These defects were reversed by Ad-PRMT5 overexpression (Fig. 5p). In vitro, high glucose similarly disrupted Complex I assembly, which was restored by PRMT5 overexpression but further aggravated by the PRMT5 inhibitor EPZ (Fig. 5q, Supplementary Fig. S2i). Importantly, the methylation-deficient Ndufs2 R118H mutant failed to rescue Complex I assembly even in the presence of PRMT5 (Fig. 5r). Collectively, PRMT5 directly interacts with Ndufs2 and catalyzes its SDMA modification at R118, which is essential for maintaining the activity and assembly integrity of Complex I in DCM.

### PRMT5-mediated SDMA modification on R118 maintains the activity of Complex I via suppressing the ubiquitin-mediated protein degradation of Ndufs2

**Fig. 6:**
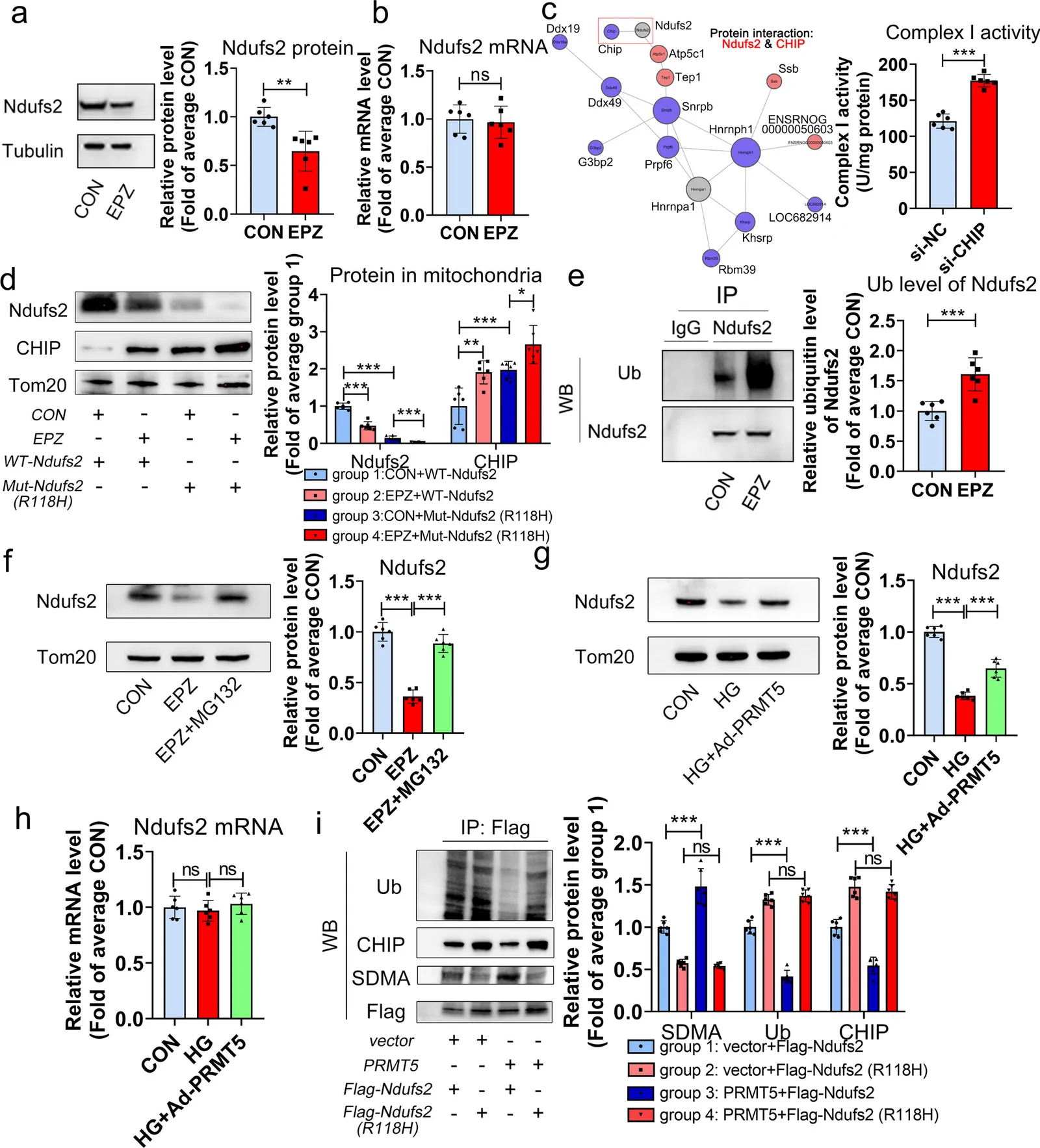
PRMT5-mediated SDMA on Ndufs2 R118 preserves Complex I activity by suppressing ubiquitin-mediated Ndufs2 degradation. **a** Western blot analysis of Ndufs2 protein levels in NRCMs with or without EPZ treatment. **b** qPCR analysis of Ndufs2 mRNA levels under the same conditions. **c** Protein–protein interaction network from IP-MS data showing Ndufs2-CHIP interaction (left). Complex I activity in NRCMs transfected with si-NC or si-CHIP (right). **d** Western blot analysis of CHIP and Ndufs2 protein levels in NRCMs transfected with WT- or R118H-mutant Ndufs2, with or without EPZ. **e** Co-IP assay showing Ndufs2 ubiquitination levels with or without EPZ treatment. **f** Western blot analysis of Ndufs2 protein levels in NRCMs treated with CON, EPZ, or EPZ + MG132. **g**–**h** Ndufs2 protein **g** and mRNA **h** levels in NRCMs under LG, HG, and HG + Ad-PRMT5 conditions. **i** Co-IP assay showing CHIP binding to Flag-tagged WT- or R118H-mutant Ndufs2, along with SDMA and ubiquitination levels of Flag-tagged Ndufs2. Tom20 was used as mitochondrial reference. Data are presented as mean ± SD; *n* = 6 biological replicates per group. Data are presented as mean ± SD. *P* values are calculated by unpaired two-tailed Student's t-test (a-c and e) or one-way ANOVA (d and f-i), *P < 0.05; **P < 0.01; ***P < 0.001, ns, no significance, *n* = 6

To explore PRMT5's regulatory mechanism on Ndufs2, we analyzed Ndufs2 expression at transcriptional and translational levels. Suppression of PRMT5's SDMA activity by EPZ or PRMT5 knockdown by si-PRMT5 reduced Ndufs2 protein without affecting mRNA levels (Fig. 6a-b, Supplementary Fig. S3a-b). High glucose (HG) treatment similarly reduced Ndufs2 protein but not mRNA (Supplementary Fig. S3c-d), suggesting the possibility of ubiquitin-mediated degradation of Ndufs2. By analyzing protein–protein interaction data derived from IP-MS sequencing (EPZ vs. CON), we identified the E3 ligase CHIP (carboxyl terminus of Hsc70-interacting protein), a mediator of ubiquitination, as a significant interactor of Ndufs2 in NRCMs (Fig. 6c). Moreover, knockdown of CHIP by si-CHIP increased the activity of Complex I, suggesting that CHIP may influence Complex I function (Fig. 6c). Further experiments showed that CHIP protein level was increased in NRCMs treated with EPZ (Fig. 6d). Co-IP assays showed that EPZ treatment, PRMT5 knockdown, or HG exposure increased Ndufs2 ubiquitination (Fig. 6e, Supplementary Fig. S3e-f), and the ubiquitin–proteasome inhibitor MG132 rescued EPZ-induced Ndufs2 reduction (Fig. 6f). Conversely, PRMT5 overexpression increased Ndufs2 protein level without affecting Ndufs2 mRNA level both in vitro and in vivo (Fig. 6g-h, Supplementary Fig. S3g–h). Moreover, PRMT5 overexpression reduced the ubiquitination level of WT-Ndufs2, whereas no significant change was observed in R118H-mutant Ndufs2 (Fig. 6i).

To identify the key ubiquitination sites of Ndufs2 regulated by PRMT5, we analyzed adjacent lysine residues of R118 and constructed Flag-tagged Ndufs2 mutants with lysine-to-arginine substitutions at K108, K122, and K127 (Flag-Ndufs2-K108R/K122R/K127R, Mut-Ndufs2). Mutagenesis of Ndufs2's adjacent lysine residues abolished EPZ-induced ubiquitination (Supplementary Fig. S3i), suggesting these lysine residues as key ubiquitination sites regulated by PRMT5-mediated SDMA.

Collectively, these findings demonstrate that PRMT5-catalyzed SDMA modification on R118 stabilizes Ndufs2 protein by antagonizing its ubiquitination-dependent degradation, thereby preserving the structural integrity and enzymatic function of Complex I.

### Serum PRMT5 is associated with DCM and shows potential as a clinical biomarker

**Fig. 7:**
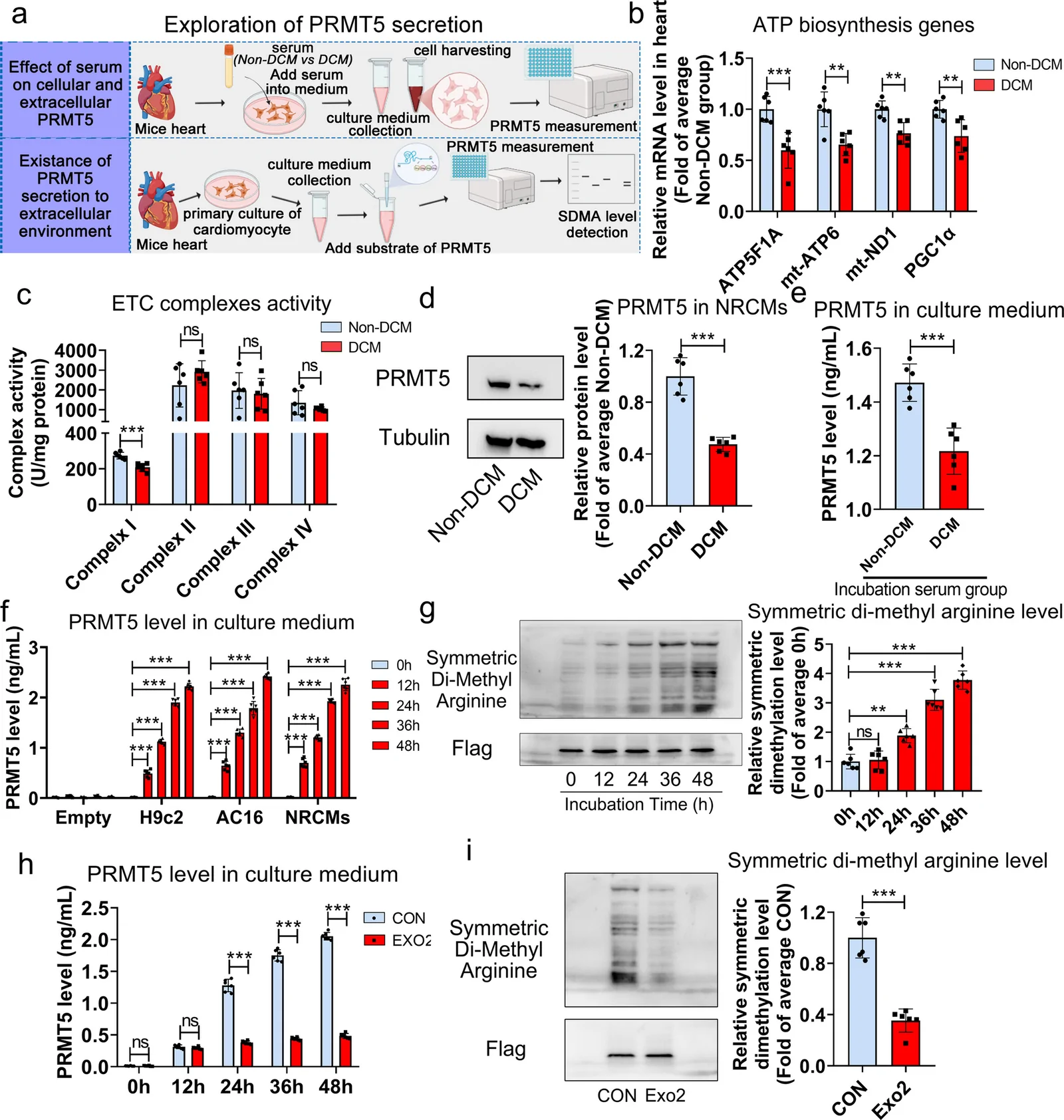
Exploration of myocardium PRMT5 excretion into serum. **a** Schematic of the study design for exploration of PRMT5 secretion. **b** qPCR analysis of ATP biosynthesis-related genes in NRCMs treated with serum from non-DCM or DCM patients. **c** Activities of mitochondrial ETC complexes I–IV in NRCMs under the same conditions. **d** Western blot analysis of PRMT5 protein levels in NRCMs. **e** ELISA detection of PRMT5 levels in NRCM culture medium. **f** Time-course PRMT5 levels in culture medium of H9c2 cells, AC16 cells, and NRCMs incubated with non-DCM or DCM serum for 0–48 h (empty, negative control). **g** Time-course SDMA levels of Flag-tagged protein in NRCM culture medium at 0–48 h. **h** Time-course PRMT5 levels in NRCM culture medium treated with solvent or Exo2 for 0–48 h (CON, negative control). **i** Western blotting assay showed the arginine SDMA level of the Flag-tagged protein in the culture medium of NRCMs treated with or without Exo2. Data are presented as mean ± SD. P values by unpaired two-tailed Student's t-test, **P < 0.01, ***P < 0.001, ns, no significance, *n* = 6

To further investigate the clinical application of PRMT5 in DCM, we conducted a series of related studies as illustrated in Fig. 7a. We found that NRCMs exposed to DCM patients’ serum exhibited downregulated ATP biosynthesis genes (ATP5F1A, mt-ATP6, mt-ND1, and PGC1α), reduced Complex I activity, as well as reduced intracellular and extracellular PRMT5 protein levels (Fig. 7b–e). Time-course experiments showed PRMT5 is secreted by cardiomyocytes (H9c2, AC16, NRCMs) in a time-dependent manner (Fig. 7f), with increasing symmetric di-methyl arginine level (Fig. 7g). Treatment with the exosome inhibitor Exo2 reduced extracellular PRMT5 and symmetric di-methyl arginine levels (Fig. 7h–i), indicating exosome-mediated PRMT5 secretion in cardiomyocytes.

**Fig. 8:**
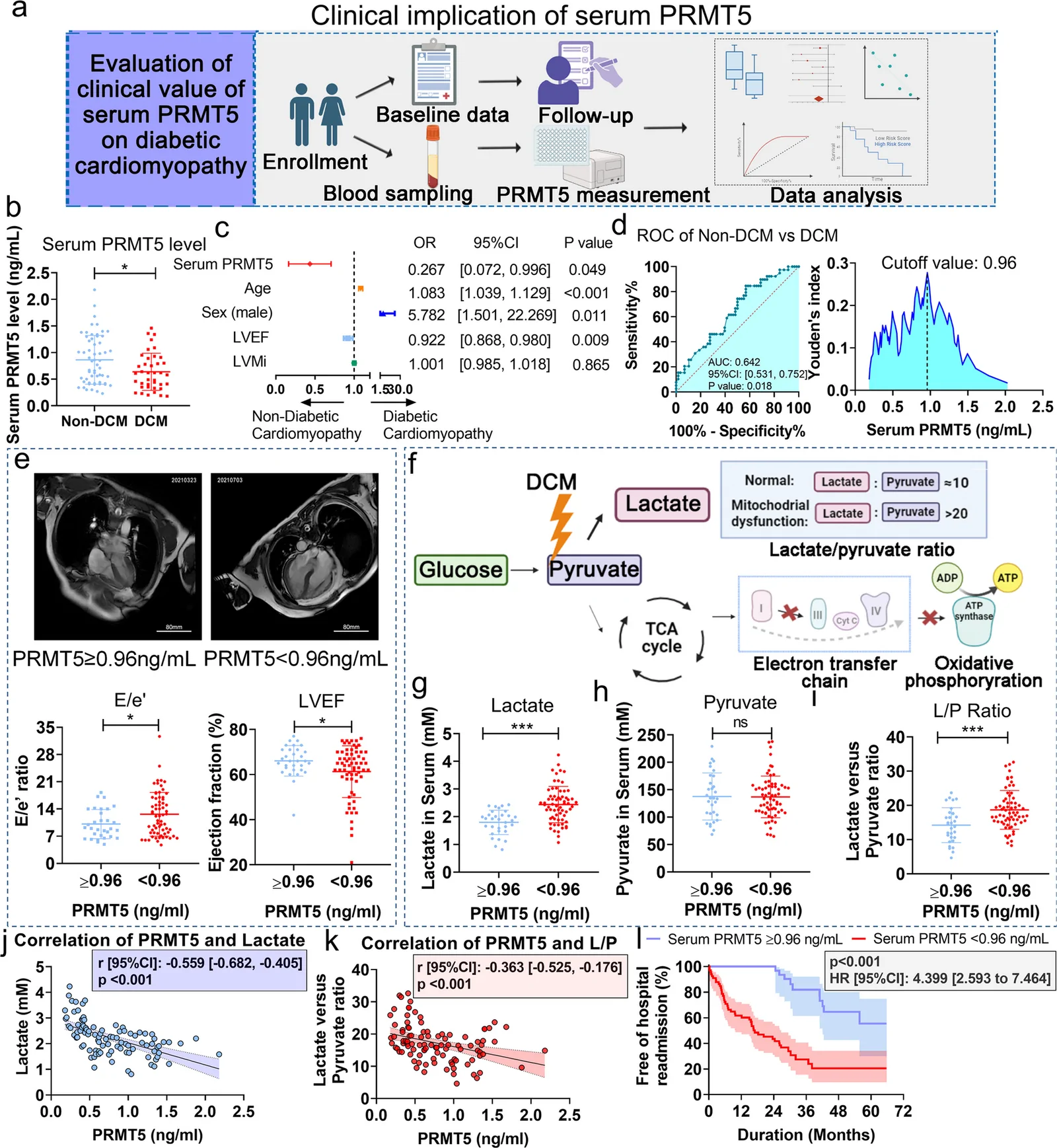
Serum PRMT5 as a potential biomarker for DCM progression. **a** Schematic of the study design for exploration of clinical implication of serum PRMT5. **b** Serum PRMT5 levels in participants with or without DCM measured by ELISA. **c** Forest plot showing serum PRMT5 as a predictor of DCM after adjustment for age and sex. OR, odds ratio; 95% CI, 95% confidence interval. **d** ROC analysis of serum PRMT5 for distinguishing non-DCM from DCM participants. Left pannel: ROC curve; right pannel: optimal cutoff determination based on maximal Youden's index. **e** E/e' ratio and LVEF by cardiac magnetic resonance in participants stratified by PRMT5 cutoff. Scale bar = 80 mm. **f** Schematic showing ETC dysfunction leading to OXPHOS inhibition and elevated lactate/pyruvate (L/P) ratio. **g**-**i** Serum lactate (**g**), pyruvate **h**, and L/P ratio (**i**) in participants stratified by PRMT5 cutoff. (**j**-**k**) Correlation of serum PRMT5 with serum lactate (**r**) or L/P ratio (**s**) by Pearson's analysis. **l** Kaplan–Meier analysis of hospital readmission risk in participants stratified by PRMT5 cutoff. Data are presented as mean ± SD (**b**, **e**, **g**-**i**). *P* values by unpaired two-tailed Student's t-test (**b**, **e**, **g**-**i**), Wald test (**c**), Pearson's correlation (**j**-**k**) or log-rank test (**l**), *n* = 58 non-DCM and 39 DCM participants (j-l), *n* = 31 (PRMT5 ≥ 0.96 ng/mL) and 66 (PRMT5 < 0.96 ng/mL) participants (e-i), *n* = 97 (**j**-**l**)

To evaluate the clinical utility of serum PRMT5 in DCM, we next compared serum PRMT5 levels between 58 participants without DCM and 39 patients with DCM; the flow chart of the study cohort is demonstrated in Fig. 8a and Fig. S4a. Baseline characteristics revealed significant differences in clinical parameters between groups: the DCM cohort was older, had elevated NT-proBNP levels, and had reduced serum PRMT5 levels (Table 1). Consistent with the diagnosis of DCM, patients in the DCM group exhibited increased E/e' ratio, elevated LVMi, and lower LVEF, indicating both systolic and diastolic dysfunction.

In addition, serum PRMT5 levels were significantly lower in DCM patients compared with non-DCM controls (Table 1, Fig. 8b). Univariate and multivariate logistic regression analysis identified serum PRMT5 as an independent protective parameter associated with DCM (Table S6, Table S7, Fig. 8c). ROC analysis demonstrated moderate diagnostic accuracy of serum PRMT5 for distinguishing DCM, with an AUC of 0.642 (95% CI: 0.531–0.752; p = 0.018) and an optimal cutoff value of 0.96 ng/mL (sensitivity = 71.8%, specificity = 58.6%) (Fig. 8d, Table S8). Stratification by this cutoff value revealed that low serum PRMT5 level (< 0.96 ng/mL) was associated with impaired diastolic and systolic cardiac function (Fig. 8e, Video 1–2). Additionally, as the L/P ratio is an established indicator of mitochondrial dysfunction, which reflects the cytosolic NADH/NAD⁺ redox state (Luengo et al. 2021), we also examined its relationship with serum PRMT5. The L/P ratio serves as a readout of cytosolic NADH/NAD⁺ redox state, with its elevation indicating compromised electron transport chain (ETC) function and mitochondrial impairment (Fig. 8f). Consistent with our mechanistic findings, participants in the low PRMT5 group showed elevated serum lactate and lactate/pyruvate (L/P) ratio (Fig. 8g–i). Pearson correlation analysis showed that serum PRMT5 was negatively correlated with lactate (r = −0.559, 95% CI: −0.682 to −0.405; *P* < 0.001) and L/P ratio (r = −0.363, 95% CI: −0.525 to −0.176; *P* < 0.001) (Fig. 8j–k). Kaplan–Meier analysis showed that low PRMT5 was associated with increased hospital readmission risk (HR = 4.399, 95% CI: 2.593–7.464; *P* < 0.001) (Fig. 8l). ROC curve analysis further supported the prognostic value of serum PRMT5 in predicting hospital readmission events within 1–5 years (Fig. S4b).

**Table 1:** Baseline characteristics of the study population

	**Non-DCM**	**DCM**	^**d**^ ***P*** ** value**
Age (y)^a^	57.77 ± 11.70	66.36 ± 16.56	0.004
Male (Total)^c^	36 (58)	31 (39)	0.087
Serum PRMT5 (ng/mL)^a^	0.86 ± 0.46	0.64 ± 0.35	0.011
NT-proBNP (pg/mL)^b^	75.04 [26.68, 126.25]	164.70 [46.34, 1217.00]	0.021
BMI (kg/m2)^a^	24.22 ± 3.01	24.33 ± 3.04	0.861
IVSTd (mm)^a^	9.93 ± 2.83	10.64 ± 2.35	0.199
LVPWd (mm)^a^	9.60 ± 1.32	9.85 ± 1.31	0.376
LVIDd (mm)^a^	47.03 ± 7.20	49.79 ± 8.55	0.089
E/e’^a^	10.45 ± 4.86	13.87 ± 5.10	0.001
HbA1c (%)^a^	5.71 ± 0.41	8.01 ± 1.62	< 0.001
LVEF (%)^a^	66.76 ± 8.60	58.49 ± 11.43	< 0.001
LVMi (g/m2)^a^	94.92 ± 31.17	109.30 ± 32.87	0.032

This table presents the baseline clinical and laboratory characteristics of the study population, including 58 non-dilated cardiomyopathy (Non-DCM) patients and 39 dilated cardiomyopathy (DCM) patients (total 97 participants; all observations represent biological replicates, with no technical replicates performed)

^a^Continuous variables are expressed as mean ± SD for normally distributed data

^b^Continuous variables are expressed as median [interquartile range] for non-normally distributed data

^c^Categorical variables are expressed as count (total number)

^d^Statistical analyses were performed using the unpaired two-tailed Student’s t-test for normally distributed continuous variables, the Mann–Whitney U test for non-normally distributed continuous variables, and the chi-square test for categorical variables to generate the reported P values. *P* < 0.05 was considered statistically significant

Collectively, these findings suggest that serum PRMT5 may have predictive value for DCM identification and correlates with cardiac function, OXPHOS/ETC activity, and readmission risk in this study cohort.

## Discussion

### Main findings of the study

DCM refers to diabetes-induced diffuse extravascular abnormalities, metabolic disorders and myocardial energy deficiency that ultimately cause cardiac dysfunction (Jankauskas et al. 2021; Nakamura et al. 2022, 23. Wu et al. 2023). Notably, multiple lines of evidence have established that mitochondrial Complex I is a primary target of dysfunction in.

diabetic heart disease. Vazquez et al. demonstrated that in STZ-induced type 1 diabetic rats, electron transport chain complex assembly was selectively impaired at Complex I, accompanied by decreased mitochondrial biogenesis and increased lysine acetylation of mitochondrial proteins (Vazquez et al. 2015). Separate work by Dabkowski et al. further showed that Complex I respiratory activity was significantly reduced specifically in the interfibrillar mitochondrial subpopulation of diabetic hearts (Dabkowski et al. 2009). Our study extends this converging evidence by revealing a previously uncharacterized post-translational regulatory layer: PRMT5 catalyzes SDMA modification at the R118 residue of Ndufs2, the catalytic core subunit of Complex I, which competitively inhibits CHIP-mediated ubiquitin-proteasomal degradation of Ndufs2 and sustains intact Complex I assembly and OXPHOS efficiency. This finding is supported by prior evidence that L-arginine supplementation mitigates DCM progression (Fiordelisi et al. 2024).

Beyond intracellular myocardial regulatory mechanisms, this work provides translational clinical insights by identifying cardiomyocyte-derived exosomal secretion of PRMT5 into peripheral circulation. The observed associations between circulating PRMT5 concentrations, DCM severity, mitochondrial dysfunction signatures, and risk of hospital readmission position serum PRMT5 as a promising circulating biomarker reflecting cardiac mitochondrial homeostasis and predicting adverse clinical outcomes. This linkage adds new layers to the evolving understanding of molecular events underlying the early stages of DCM (Benchea et al. 2025).

### PRMT5-mediated SDMA modification of Ndufs2 at R118: mechanism and specificity

PRMT5 is a type II PRMT enzyme that catalyzes SDMA modification on substrates by transferring methyl groups from SAM to arginine residues. Deregulation of PRMT5 has been implicated in the pathogenesis of various diseases, including diabetes, cardiovascular diseases, and aging (Jia et al. 2020; Tan et al. 2019; Chauhan et al. 2023). Cumulative evidence confirms the overall effects of PRMT5 are largely dependent on its enzymatic activity (Cheung et al. 2007; Kim et al. 2016; Le Romancer et al. 2010; Mitchell et al. 2009). Consistently, our data confirm that PRMT5 is essential for maintaining ETC Complex I function by the evidences that both pharmacological inhibition (EPZ) and genetic knockdown of PRMT5 impaired Complex I activity and integrity, while catalytically inactive PRMT5 mutant failed to rescue Ndufs2 SDMA modification or enhance Complex I activity.

Furthermore, multiple lines of evidence support the on-target specificity of EPZ in our experimental system. First, PRMT5 inhibition exclusively disrupted cellular SDMA pools without triggering compensatory upregulation of alternative arginine methyltransferases or shifts in global ADMA landscapes. Second, pharmacological inhibition by EPZ, genetic inhibition by si-PRMT5 and catalytic mutation produced similar phenotypes, including decreased Complex I activity and impaired mitochondrial function.

PRMT5 exhibits dynamic subcellular trafficking under cardiac pathological stress. Previous phenylephrine-induced cardiomyocyte hypertrophy models documented nuclear-to-cytoplasmic translocation of PRMT5 to modulate cytoplasmic substrate methylation (Chen et al. 2014). Extrapolating this regulatory pattern, we hypothesize chronic hyperglycemia in DCM drives comparable PRMT5 cytoplasmic redistribution, enabling physical interaction with Ndufs2 localized to the inner mitochondrial membrane. While direct evidence documenting high-glucose-triggered PRMT5 mitochondrial translocation remains limited in existing literature, our immunofluorescence and Co-IP data support functional coupling between cytoplasmic PRMT5 and mitochondrial Ndufs2 under diabetic stress. Future subcellular fractionation and live-cell fluorescence tracking experiments will be required to definitively validate this trafficking hypothesis.

In this study, Ndufs2 was selected as a crucial PRMT5 substrate for the following reasons: (1) IP-MS profiling identified Ndufs2 as the second-ranked protein with drastically suppressed SDMA abundance upon PRMT5 inhibition. (2) KEGG pathway overrepresentation further linked PRMT5-dependent SDMA substrates to two DCM-relevant biological modules: oxidative phosphorylation and ubiquitin-mediated proteolysis, with Ndufs2 serving as the shared core effector protein bridging both pathways. (3) As the catalytic Fe-S cluster scaffold of Complex I, Ndufs2 loss completely abolishes NADH electron transfer and depletes intracellular ATP reserves (Zhang et al. 2024; Tuppen et al. 2010; Scheffler 2015). (4) The R118 SDMA site resides within the conserved Fe-S binding domain (amino acids 34–463) of Ndufs2, implying methylation at this residue directly modulates electron transport efficiency. Recombinant in vitro interaction and methylation assays confirm PRMT5 directly binds Ndufs2 in an R118-dependent manner, and catalytically impaired PRMT5 cannot install SDMA marks on wild-type Ndufs2. Reciprocally, ectopic expression of methylation-deficient Ndufs2-R118H abrogates the Complex I-rescuing effect of PRMT5 overexpression under hyperglycemic conditions, verifying R118 as the functionally indispensable modification locus.

Notably, Ndufs2 arginine methylation is also governed by the assembly factor NDUFAF7, which acts through a spatially distinct residue and fulfills divergent physiological roles during cardiac development versus metabolic stress. Rhein et al. demonstrated that NDUFAF7 catalyzes SDMA of NDUFS2 at Arg-85, a modification essential for stabilizing the 400-kDa subcomplex during Complex I assembly (Rhein et al. 2013). Zurita Rendón et al. further showed that NDUFAF7 deficiency leads to rapid degradation of ND1 (the Complex I assembly seed subunit) and reduced steady-state levels of NDUFS2 and other Complex I subunits, establishing that this methylation is indispensable for Complex I biogenesis and vertebrate development (Zurita Rendón et al. 2014). Our findings distinguish PRMT5 from NDUFAF7 by defining a stress-responsive regulatory pathway: PRMT5-mediated R118 methylation preserves pre-assembled mature Complex I against ubiquitin-dependent breakdown in the hyperglycemic diabetic myocardium, whereas NDUFAF7-R85 methylation governs de novo Complex I formation during developmental stages. These two methyltransferases thus operate in complementary temporal and spatial windows to maintain mitochondrial respiratory chain homeostasis.

Among remaining type II PRMTs capable of SDMA catalysis, PRMT9 is functionally irrelevant to Ndufs2 regulation in cardiomyocytes (Hadjikyriacou et al. 2015). Global SDMA profiling of PRMT5 knockout fibroblasts demonstrates PRMT5 contributes approximately 95% of total cellular SDMA, establishing its dominant enzymatic role (Gao et al. 2019). Independent protein interaction screening ruled out PRMT9 as a binding partner of Ndufs2, eliminating its compensatory regulatory capacity on Ndufs2 methylation (Fig. S5).

Mechanistically, PRMT5-mediated R118 methylation creates steric hindrance that blocks recruitment of the E3 ubiquitin ligase CHIP to Ndufs2. Global ubiquitin proteome profiling identified CHIP as the primary ubiquitination effector targeting Ndufs2 when PRMT5 SDMA activity is lost. Biochemical assays confirm PRMT5 depletion enhances CHIP-Ndufs2 binding and elevates total Ndufs2 ubiquitination, while PRMT5 overexpression reverses this phenotype exclusively for wild-type Ndufs2. Site-directed mutagenesis of three lysine residues proximal to R118 (K108R/K122R/K127R) fully abrogates EPZ-induced Ndufs2 ubiquitination, identifying these residues as the primary CHIP-mediated ubiquitination docking sites modulated by R118 SDMA. Consistent with this regulatory cascade, ubiquitin-dependent proteolysis ranked second in KEGG enrichment of PRMT5-inhibited SDMA substrates, reinforcing the methylation-ubiquitination crosstalk as a core PRMT5 effector pathway.

All rescue phenotypes observed following adenoviral PRMT5 overexpression are attributed to PRMT5-specific biological activity, rather than off-target viral vector toxicity. Parallel in vivo and in vitro vector control cohorts confirmed that empty viral/plasmid delivery exerted no independent effects on cardiac hypertrophy, mitochondrial respiration or Complex I stability, excluding vector-related experimental artifacts (Supplementary Table S9, S10).

### Cell-type specificity of the PRMT5/Ndufs2 axis in cardiomyocytes

After delineating the molecular crosstalk between PRMT5 and Ndufs2, we further dissect why this regulatory cascade exerts cell-specific effects confined to cardiomyocytes. The PRMT5-Ndufs2 protective cascade exhibits cardiomyocyte-specific functional priority, stemming from unique energetic and metabolic properties of cardiac muscle cells. Cardiomyocytes contain 30–40% mitochondrial volume and generate over 90% of ATP via OXPHOS, creating an absolute reliance on unimpaired Complex I function to sustain continuous contractile activity (Rudokas et al. 2024; Murphy and Liu 2023). PRMT5-dependent stabilization of Ndufs2 safeguards rate-limiting Complex I electron transfer under hyperglycemic stress, a non-redundant survival mechanism for high-energy-demand cardiomyocytes. By contrast, fibroblasts and hepatocytes maintain lower mitochondrial density and rely heavily on glycolysis (20–50% of total ATP production) to offset transient Complex I dysfunction (Lunt and Vander Heiden 2011; Yang et al. 2015; Ainscow and Brand 1999). Thus, these cell types possess redundant metabolic bypass pathways that negate the requirement for PRMT5-mediated Ndufs2 protection.

PRMT5 also displays cell-type-specific substrate selectivity across somatic tissues. In non-cardiac cell lineages, PRMT5 predominantly methylates nuclear histones and transcription factors to govern proliferation and epigenetic remodeling. In mature cardiomyocytes, cytoplasmic PRMT5 preferentially targets mitochondrial substrates including Ndufs2 to sustain respiratory chain integrity, explaining why loss of PRMT5 selectively precipitates mitochondrial failure in cardiac tissue rather than other organs (Liu et al. 2015; Litzler et al. 2019).

It is also important to note that the cardioprotective effects of PRMT5 are unlikely to be secondary to improved systemic metabolism, as our previous study demonstrated direct anti-hypertrophic activity of PRMT5 in non-metabolic cardiac stress models including isoproterenol-induced and spontaneously hypertensive rats (Cai et al. 2020). In the present study, we further identify a potential cardiomyocyte-intrinsic mechanism whereby PRMT5 preserves mitochondrial Complex I function via Ndufs2 R118 methylation under diabetic conditions.

Collectively, the PRMT5/Ndufs2 axis constitutes a cardiomyocyte-exclusive protective cascade, which may provide a cell-specific therapeutic target for energy-deficient diabetic cardiac injury.

### Limitations of the study

Elucidating the interaction among PRMT5, Ndufs2, and CHIP is important for dissecting the regulatory cascade in DCM. However, given the mutually competitive nature between PRMT5-mediated SDMA and CHIP-catalyzed ubiquitination, which may lead to mutually exclusive interactions, triple co-IP or GST pull-down assays are unlikely to detect concurrent binding and may not yield definitive evidence. Despite this technical limitation, four independent lines of indirect evidence validate the competitive regulatory relationship. First, protein–protein interaction analysis from IP-MS data identified CHIP as the major E3 ligase associated with Ndufs2 upon PRMT5 suppression. Second, PRMT5 inhibition significantly enhanced CHIP-Ndufs2 binding and Ndufs2 ubiquitination, whereas PRMT5 overexpression reduced their interaction, indicating PRMT5 antagonizes CHIP recruitment. Third, R118 methylation-deficient Ndufs2 cannot evade CHIP recruitment even with excess PRMT5. Fourth, mutation of CHIP-targeted lysine residues markedly attenuated Ndufs2 ubiquitination, supporting CHIP as the key E3 ligase for Ndufs2. Further orthogonal experimental approaches such as proximity ligation assay will be required to visualize dynamic shifts in Ndufs2 binding partners under fluctuating PRMT5 activity.

It is reported that post-translational modification of one amino acid residue can affect adjacent residues (Fulton et al. 2022; Zhao et al. 2021). According to Uniprot Database (Supplementary Uniprot data), K108, K122 and K127 are the closest residues to R118 and recognizable by ubiquitin-mediated degradation enzymes. In this study, although K108, K122 and K127 were identified as critical ubiquitination residues modulated by R118 SDMA, this study does not resolve which single lysine residue serves as the primary CHIP ubiquitination acceptor site. Site-specific single-lysine mutagenesis will be necessary in future work to pinpoint the dominant ubiquitination locus regulated by PRMT5-mediated methylation crosstalk.

Several limitations of the clinical study should also be acknowledged. First, this was a single-center study with a relatively modest sample size, which may limit the statistical power and generalizability of our findings. Larger, multi-center prospective cohorts with serial longitudinal blood sampling are needed to validate the diagnostic and prognostic value of serum PRMT5 for DCM. Second, the observational cross-sectional design cannot establish causal directionality between low circulating PRMT5 and DCM progression. It remains undetermined whether diminished cardiac PRMT5 secretion acts as an upstream pathogenic driver of mitochondrial injury, or merely a secondary biomarker of established myocardial damage. Thus, long-term serial biomarker tracking in prediabetic patients will resolve this temporal relationship. Third, the specificity of serum PRMT5 for DCM requires further investigation. PRMT5 is ubiquitously expressed across epithelial, immune and metabolic organs, so future studies must enroll patients with non-diabetic heart failure, renal disease and metabolic syndrome to quantify circulating PRMT5’s specificity for DCM. Fourth, while our preclinical data support PRMT5 as a potential therapeutic target, translating these findings from rodent models and cell culture to human patients presents significant challenges. The safety and efficacy of PRMT5-targeted therapies for DCM remain to be evaluated in clinical settings. Finally, the diagnostic performance of serum PRMT5 (AUC = 0.642) is moderate, and it may serve best as part of a multi-biomarker panel rather than a standalone diagnostic tool. Further research is needed to determine whether combining PRMT5 with other biomarkers can improve diagnostic accuracy for DCM.

### Implications and advantages of serum PRMT5 in diagnosis of DCM

Serum PRMT5 may offer complementary value to established DCM biomarkers including NT-proBNP and cTnI, both of which have well-documented limitations in specific clinical scenarios. Specifically, NT-proBNP reflects late-stage ventricular wall stress and irreversible myocardial remodeling, yet it exhibits low specificity for diabetes-specific cardiac injury (Gao et al. 2022; Ammar et al. 2025; Landolfo et al. 2024). As a marker reflecting end-stage cardiac decompensation, NT-proBNP cannot quantify upstream mitochondrial dysfunction or monitor therapeutic efficacy of mitochondrial-targeted DCM treatments. On the other hand, cardiac troponins (cTnI/cTnT) are sensitive for myocardial necrosis and exclude acute coronary events in DCM diagnosis (Park et al. 2017), but they lack specificity for chronic, subclinical mitochondrial damage that develops prior to overt heart failure (Ianoș et al. 2024). Consequently, neither of these biomarkers can deliver direct readouts of electron transport chain Complex I activity, the initiating pathogenic lesion in DCM.

Serum PRMT5 possesses two unique translational advantages absent from conventional cardiac biomarkers. First, it functions as a mechanistic circulating readout of myocardial mitochondrial health. Extensive metabolomic research establishes elevated lactate and lactate/pyruvate (L/P) ratios as validated surrogates for impaired cardiac OXPHOS and NADH redox imbalance (Fujii et al. 2014; Guo et al. 2025). Our cohort correlation analyses confirm serum PRMT5 concentrations negatively correlate with circulating lactate and L/P ratios, directly linking peripheral PRMT5 levels to cardiac Complex I functional status, a mechanistic linkage absent from NT-proBNP and troponin measurements. Second, PRMT5 integrates diagnostic and prognostic value with DCM specificity. After adjustment for age and sex, circulating PRMT5 served as an independent protective predictor of DCM (OR = 0.266, 95% CI = 0.076–0.933). Its moderate diagnostic discrimination capacity (AUC = 0.642) aligns with most single metabolic-derived circulating markers for diabetic heart disease. Long-term follow-up further demonstrated that patients with reduced serum PRMT5 carried a 4.4-fold higher risk of recurrent hospital admission (HR = 4.399), highlighting its prognostic stratification value.

Collectively, serum PRMT5 shows preliminary value as a mechanistic biomarker that reflects mitochondrial dysfunction in DCM, with potential utility in diagnosis and prognosis assessment. Further validation in larger cohorts is warranted to determine its clinical role in DCM.

## Conclusion

This study demonstrates that PRMT5 protects against DCM by SDMA modification on Ndufs2 at residue R118. This modification antagonizes the ubiquitin-proteasomal degradation of Ndufs2, thereby preserving the activity of Complex I in the ETC, as well as maintaining OXPHOS and ATP production (Graphical Abstract). Clinically, PRMT5 shows potential as a target for developing novel diagnostic and therapeutic approaches for DCM. Further studies are warranted to validate these findings and explore their clinical translational potential.

## Supplementary Information

Supplementary Material 1.

Supplementary Material 2.

Supplementary Material 3.

Supplementary Material 4.

Supplementary Material 5.

Supplementary Material 6.

Supplementary Material 7.

Supplementary Material 8.

Supplementary Material 9.

Supplementary Material 10.

Supplementary Material 11.

Supplementary Material 12.

Supplementary Material 13.

Supplementary Material 14.

## Data Availability

The datasets analyzed during this study are included in this article and its supplementary information files.

## Abbreviations

AUC: Area under the curve
BMI: Body mass index
95%CI: 95% Confidence Interval
Co-IP: Co-immunoprecipitation
CSA: Cell surface area
DCM: Diabetic cardiomyopathy
EF: Ejection fraction
E/e’: Mitral inflow velocity versus mitral annular velocity at early-diastole state
ETC: Electron transport chain
FBG: Fasting blood glucose
H&E: Hematoxylin and eosin
HFD: High-fat diet
HW: Heart weight
HW/TL: Heart weight versus tibia length
IP-MS: Mass spectrometry
ITT: Insulin tolerance test
IVSTd: Interventricular septum at diastole state
LC-MS: Liquid chromatography-tandem mass spectrometry
L/P ratio: Serum lactate versus pyruvate ratio
LVID: Left ventricular internal dimension at diastole state
LVMi: Left ventricular mass index
LVPW: Left ventricular posterior wall thickness
Ndufs2: NADH dehydrogenase [ubiquinone] iron-sulfur protein 2
NRCMs: Neonatal rat cardiomyocytes
OGTT: Oral glucose tolerance test
OR: Odds ratio
OXPHOS: Oxidative phosphorylation
PRMT5: Protein arginine methyltransferase 5
Q-PCR: Quantitative polymerase chain reaction
ROC: Receiver-operating characteristic curve
SDMA: Symmetric di-methylation of arginine
SPF: Specific pathogen free
STZ: Streptozotocin
