# Supplementary material for: Targeting PRMT5 alleviates diabetic cardiomyopathy: mechanisms involve Ndufs2 R118 dimethylation-mediated complex I protection and serum PRMT5 as a biomarker
Source: Mol Med. 2026 Aug 13;32:213. doi: 10.1186/s10020-026-01594-2 (PMC13749988; doi:10.1186/s10020-026-01594-2)
Supplement: Supplementary file 1 [file 10020_2026_1594_MOESM11_ESM.docx]

**SUPPLEMENTARY FIGURES**

Targeting PRMT5 Alleviates Diabetic Cardiomyopathy: Mechanisms Involve Ndufs2 R118 Dimethylation-Mediated Complex I Protection and Serum PRMT5 as a Biomarker

Sidong Cai^a^, Mengqi Su^a^, Jing Li^a^, Yang Yu^b^, Min Wu^a^, Mingya Liu^a^, Run Wang^a^, Jie Huang^a,c^, Qingxia Lai^a^, Cong Chen^*a^, Kai-Hang Yiu^*a,d^

^a^ Division of Cardiology, Department of Medicine, The University of Hong Kong-Shenzhen Hospital, Shenzhen, China;

^b^ Department of Ultrasound, Yantian District People's Hospital, Shenzhen, China

^c^ Shenzhen University Health Science Center, Shenzhen, China

^d^ Department of Cardiology, The University of Hong Kong, Queen Mary Hospital, Hong Kong, China.

* Correspondence to: Prof. Cong Chen, email: chenc6@hku-szh.org;

Prof. Kai-Hang Yiu, email: khkyiu@hku.hk

Fig. S1

**Fig. S1 Echocardiographic parameters of mice at week 12 and validation of the knockdown efficiency of si-PRMT5 and overexpression effect of PRMT5 plasmid in cardiomyocytes.** (a-c) IVSTd, LVIDd and LVEF were measured by echocardiography in mice of group 1-4 at week 12. Group 1: CON+Ad-vector group; group 2: Ad-vector+HFD/STZ group; group 3: CON+Ad-PRMT5 group; group 4: Ad-PRMT5+HFD/STZ group. (d) mRNA level of PRMT5 in heart, lung, liver and kidney of Ad-vector or Ad-PRMT5 group were measure by q-PCR. (e) si-NC or si-PRMT5 are transfected into cardiomyocytes in 6-well dishes for 48h, PRMT5 protein level are measured by Western blotting. (f) vector or PRMT5 plasmid are transfected into cardiomyocytes in 6-well dishes for 48h, PRMT5 protein level are measured by western blotting. (g) Total arginine symmetric di-methylation level in cardiomyocytes treated without or with different concentrations of PRMT5 enzymatic inhibitors EPZ015666 are measured by western blotting. Data are presented as mean ± SD. P values were calculated by one-way ANOVA (a-c) or unpaired two-tailed Student's t-test (d) *p < 0.05, **p < 0.01, ***p < 0.001, ns, no significance, n = 6.

Fig. S2

**Fig. S2 PRMT5-mediated SDMA modification of Ndufs2 at R118 regulates Complex I assembly and mitochondrial function in cardiomyocytes.** (a) The interaction between Flag‑tagged Ndufs2 (wild‑type and R118H mutant) and GST‑tagged PRMT5 (wild‑type and catalytically inactive G367A/R368A double mutant), as well as the levels of SDMA and ubiquitin level of Flag‑tagged Ndufs2 in NRCMs were measured by Co-IP assay. (b) Cardiomyocytes in 6-well dishes are treated with negative control solution (CON) or PRMT5 enzymatic inhibitor EPZ for 24h, PRMT1, 2, 3, 4, 6, 7, 8 and 9 protein level are measured by Western blotting. (c) Cardiomyocytes in 6-well dishes are treated with negative control solution (CON) or PRMT5 enzymatic inhibitor EPZ for 24h, AMDA level are measured by Western blotting. (d) ADMA level in cardiomyocytes transfected with si-NC or with si-PRMT5 are measured by western blotting. (e) ELISA assay showed activity of Complex II, III and IV in NRCMs treated with or without EPZ. (f) ELISA assay showed activity of Complex II, III and IV in NRCMs transfected with si-NC or si-PRMT5. (g) ELISA assay showed activity of Complex II, III and IV in NRCMs in LG, HG, HG+PRMT5 group. (h) ELISA assay showed the ATP level in NRCMs transfected with wild type Ndufs2 plasmid (Ndufs2 group), PRMT5 and wild type Ndufs2 plasmid (PRMT5+Ndufs2 group), arginine mutagenous Ndufs2 plasmid (Ndufs2 (R118H) group) and PRMT5 and arginine mutagenous Ndufs2 plasmid (PRMT5+Ndufs2 (R118H) group) in both low glucose culture medium and high glucose culture medium. (i) BN‑PAGE analysis was performed to analyze Complex I integrity in NRCMs transfected with WT-Ndufs2 or R118H mutant-Ndufs2 with or without PRMT5. Tom20 was used as mitochondrial reference protein. Data are presented as mean ± SD. P values were calculated by unpaired two-tailed Student's t-test (b-f) or one-way ANOVA (g-h), ***p < 0.001, ns, no significance, n = 6.

Fig. S3

**Fig. S3 Validation of the knockdown efficiency of si-PRMT5 and overexpression effect of PRMT5 plasmid in cardiomyocytes.** (a) Western blotting assay was used to measure the protein level of Ndufs2 in NRCMs transfected with si-NC or si-PRMT5. (b) Q-PCR was performed to detect the mRNA level of Ndufs2 in NRCMs transfected with si-NC or si-PRMT5. (c) Western blotting assay was used to measure the protein level of Ndufs2 in NRCMs cultured in culture medium with low glucose (LG) or high glucose (HG). (d) Q-PCR was performed to detect the mRNA level of Ndufs2 in NRCMs cultured in culture medium with LG or HG. (e) Co-IP assay was performed to determine the ubiquitination level of Ndufs2 in NRCMs transfected with or si-NC or si-PRMT5. (f) Co-IP assay was performed to determine the SDMA and ubiquitination level of Ndufs2 in NRCMs in culture medium with LG or HG. (g) Q-PCR was performed to detect the mRNA level of Ndufs2 in heart tissue of mice in CON group, HFD/STZ group or HFD/STZ+Ad-PRMT5 group. (h) Western blotting was performed to detect the protein level of Ndufs2 in heart tissue of mice in CON group, HFD/STZ group or HFD/STZ+Ad-PRMT5 group. Tom20 was used as mitochondrial reference protein. (i) Flag-tagged Ndufs2 plasmid or Flag-tagged Ndufs2 (K108R, K122R, K127R) are transfected into cardiomyocytes in 6-well dishes for 48h, accompanied with or without EPZ. The CHIP and total ubiquitin level are measured by western blotting. Data are presented as mean ± SD. P values by unpaired two-tailed Student's t-test (a-f) or one-way ANOVA (g-i), ***p < 0.001, ns, no significance, n = 6.

Fig. S4

**Fig. S4 Flow chart of the study cohort and ROC curve of 1 to 5 year hospital readmission of study cohort.** (a) Flow chart of the study cohort. (b) ROC curve demonstrates the sensitivity and specificity of serum PRMT5 in discriminating non-readmission and readmission in 1 to 5 years. Results are demonstrated as AUC and 95% CI. Statistical significance was determined by using ROC curve analysis. N=97 biological replicates.

Fig. S5

**Fig. S5 PRMT9 protein did not bind to Ndufs2.** The PRMT9 protein level in proteins enriched by anti-Ndufs2 antibody was demonstrated by Co-immunoprecipitation assay.
