# Supplementary material for: Targeting PRMT5 alleviates diabetic cardiomyopathy: mechanisms involve Ndufs2 R118 dimethylation-mediated complex I protection and serum PRMT5 as a biomarker
Source: Mol Med. 2026 Aug 13;32:213. doi: 10.1186/s10020-026-01594-2 (PMC13749988; doi:10.1186/s10020-026-01594-2)
Supplement: Supplementary file 2 [file 10020_2026_1594_MOESM12_ESM.docx]

**SUPPLEMENTARY TABLES**

Targeting PRMT5 Alleviates Diabetic Cardiomyopathy: Mechanisms Involve Ndufs2 R118 Dimethylation-Mediated Complex I Protection and Serum PRMT5 as a Biomarker

Sidong Cai^a^, Mengqi Su^a^, Jing Li^a^, Yang Yu^b^, Min Wu^a^, Mingya Liu^a^, Run Wang^a^, Jie Huang^a,c^, Qingxia Lai^a^, Cong Chen^*a^, Kai-Hang Yiu^*a,d^

^a^ Division of Cardiology, Department of Medicine, The University of Hong Kong-Shenzhen Hospital, Shenzhen, China;

^b^ Department of Ultrasound, Yantian District People's Hospital, Shenzhen, China

^c^ Shenzhen University Health Science Center, Shenzhen, China

^d^ Department of Cardiology, The University of Hong Kong, Queen Mary Hospital, Hong Kong, China.

*Correspondence to: Prof. Kai-Hang Yiu, email: khkyiu@hku.hk;

Prof. Cong Chen, email: chenc6@hku-szh.org.

**Table S1. Antibodies**

| **Antibody** | **Catalog number** | **Company** | **Source** | **Dilution** |
| --- | --- | --- | --- | --- |
| β-MHC | GTX100713-S | GeneTex | mouse | 1:1000 |
| ANF | AF7608 | Beyotime | rabbit | 1:1000 |
| PRMT5 | 109451 | Abcam | rabbit | 1:1000 |
| Tubulin | 15115S | Cell Signaling Technology | rabbit | 1:1000 |
| IgG | #2729 | Cell Signaling Technology | rabbit | 1:1000 |
| IgG | A0192 | Beyotime | mouse | 1:1000 |
| Ndufs2 | Ab192022 | Abcam | rabbit | 1:1000 |
| Symmetric Di-Methyl Arginine | PTM-617RM | PTM Biolabs | rabbit | 1:1000 |
| Flag | F1804 | Sigma | mouse | 1:1000 |
| Ub | ab122 | Abcam | rabbit | 1:1000 |
| PRMT9 | ab101013 | Abcam | rabbit | 1:1000 |

**Table S2. Primers of mRNA**

| **Primer** | **Sequence** |
| --- | --- |
| β-actin | Forward:5'- ACAACCTTCTTGCAGCTCCTC-3' |
|  | Reverse:5'-CTGACCCATACCCACCATCAC-3' |
| ANF | Forward:5'- CGTCTTGGCCTTTTGGCTTC-3' |
|  | Reverse:5'-GGTGGTCTAGCAGGTTCTTGAAA-3' |
| BNP | Forward:5'- GTTTGGGCTGTAACGCACT-3' |
|  | Reverse:5'-TCACTTCAAAGGTGGTCCCAG-3' |
| β-MHC | Forward:5'- AGCCTCAGCAGAGGAGTACA-3' |
|  | Reverse:5'-GGCTGAGCCTTGGATTCTCA-3' |
| ATPase | Forward:5'- TGCTCCGACAAGACTGGAAC-3' |
|  | Reverse:5'- GTCAAAGGAGACCCCACTCTG-3' |
| CPT1a | Forward:5'- ATTCCAGGAGAGTGCCAGGA-3' |
|  | Reverse:5'- AGCCTTTGCCGAAAGAGTCA-3' |
| PRMT5 | Forward:5'- TAGGAGTCGTCCCTTTCCAC-3' |
|  | Reverse:5'- AGAGGAAATCAAACCCCTGCT-3' |
| CPT1b | Forward:5'- TCGAGTTCAGAAACGAACGC-3' |
|  | Reverse:5'- ACCCCTTATGCCTGTGAACTG-3' |
| CPT1c | Forward:5'- TTTCTGTATGTCACCCCCACAC-3' |
|  | Reverse:5'- CATCAGCAAGGTCGGTGGGA-3' |
| UCP1 | Forward:5'- ATCTCGGCTGGCTTGATGAC-3' |
|  | Reverse:5'- TAGGAGTCGTCCCTTTCCAC-3' |
| Ndufs2 | Forward:5'- AGGGCTACCAAGTTCCTCCA-3' |
|  | Reverse:5'- AAGTACACCCCAAACTCGCC-3' |

**Table S3. Plasmid and small interference RNA**

| **Plasmid** | **Company** | **Location** | **Catelog Number** |
| --- | --- | --- | --- |
| Flag tagged Wild type PRMT5 plasmid | Sino Biological | Suzhou, China | PRMT5-WT |
| Flag tagged mutant type PRMT5 plasmid  (R367A, R368A) | Sino Biological | Suzhou, China | PRMT5-mut |
| Flag tagged PcDNA3.1+ vector | Sino Biological | Suzhou, China | G107083-1 |
| Flag tagged Wild type Ndufs2 plasmid | Sino Biological | Suzhou, China | Ndufs2-WT |
| Flag tagged mutant type PRMT5 plasmid  (R118H) | Sino Biological | Suzhou, China | Ndufs2-mut |
| **siRNA** | **Company** | **Target transcription** | **Catelog Number** |
| si-PRMT5 | Guangzhou RiboBio Co., Ltd. | NM_001108867.2 | siBDM1999A |
| **Adenovirus** | **Company** | **Target transcription** | **Catelog Number** |
| Ad-PRMT5 | WZ Biosciences Inc. | NM_001039619 | VH863059 |

**Table S4. Reagents**

| **Reagent** | **Company** | **Catalog** |
| --- | --- | --- |
| Lipofectamine 2000 | Invitrogen | 11668019 |
| DAPI | Oneshine | FS1206-10mL |
| Anti-mouse IgG, HRP-linked secondary antibody | Cell Signaling Technology | #7076P2 |
| Anti-rabbit IgG, HRP-linked secondary antibody | Cell Signaling Technology | #7074P2 |
| rhodamine-phalloidin | GuangZhou Jiayan Biotech Co.,Ltd | 49734ES75 300T |
| protein A/G beads | ThermoFisher | 20421 |
| RIPA lysis buffer | Beyotime | P0013B |
| BCA protein assay kit | ThermoFisher | 23225 |
| Trizol | Invitrogen | 15596018 |
| RevertAid First Strand cDNA Synthesis Kit | ThermoFisher | K1622 |
| THUNDERBIRD™ SYBR qPCR Mix | Toyobo | 622500 |
| Formaldehyde solution | Guangzhou Chemical Reagent Factory | 50-00-0 |
| IP lysis buffer | Beyotime | P0013 |

**Table S5. Drugs**

| **Drug** | **Catalog number** | **Company** |
| --- | --- | --- |
| EPZ015666 | HY-12727 | MCE |
| Exo2 | HY-137975 | MCE |
| MG132 | S2619 | Selleck |

**Table S6. Univariable logistic regression analysis**

| Parameter | OR | 95% CI | P value |
| --- | --- | --- | --- |
| Age | 1.046 | 1.013-1.079 | **0.005** |
| Sex | 2.368 | 0.924-6.068 | **0.073** |
| Serum PRMT5 | 0.253 | 0.085-0.756 | **0.014** |
| NT-proBNP | 1 | 1.000-1.000 | 0.766 |
| LVEF | 0.926 | 0.882-0.973 | **0.002** |
| BMI | 1.012 | 0.884-1.159 | 0.859 |
| IVSD | 1.108 | 0.944-1.300 | 0.211 |
| LVDD | 1.049 | 0.990-1.111 | 0.108 |
| LVPWD | 1.152 | 0.844-1.574 | 0.373 |
| HbA1c | 1.574 | 0.010-3.352 | 0.969 |
| LVMi | 1.014 | 1.001-1.028 | **0.040** |

**Table S7. Multivariable logistic regression analysis**

| Parameter | OR | 95% CI | P value |
| --- | --- | --- | --- |
| Age | 1.083 | 1.039-1.129 | **<0.001** |
| Sex | 5.782 | 1.501-22.269 | **0.011** |
| Serum PRMT5 | 0.267 | 0.072-0.996 | **0.049** |
| LVEF | 0.922 | 0.868-0.980 | **0.009** |
| LVMi | 1.001 | 0.985-1.018 | **0.865** |

**Table S8. Youden’s index and cutoff of serum PRMT5**

| **Serum PRMT5 cutoff**  **(ng/mL)** | **Sensitivity%** | **Specificity%** | **^a^Youden’s index** |
| --- | --- | --- | --- |
| < 0.1850 | 2.564 | 100.0 | 0.02564 |
| < 0.1950 | 5.128 | 100.0 | 0.05128 |
| < 0.2150 | 7.692 | 100.0 | 0.07692 |
| < 0.2350 | 10.26 | 98.28 | 0.0854 |
| < 0.2450 | 12.82 | 98.28 | 0.111 |
| < 0.2600 | 15.38 | 98.28 | 0.1366 |
| < 0.2850 | 15.38 | 96.55 | 0.1193 |
| < 0.3100 | 15.38 | 93.10 | 0.0848 |
| < 0.3250 | 17.95 | 93.10 | 0.1105 |
| < 0.3350 | 20.51 | 93.10 | 0.1361 |
| < 0.3450 | 20.51 | 89.66 | 0.1017 |
| < 0.3650 | 25.64 | 89.66 | 0.153 |
| < 0.3900 | 25.64 | 87.93 | 0.1357 |
| < 0.4050 | 30.77 | 84.48 | 0.1525 |
| < 0.4150 | 33.33 | 81.03 | 0.1436 |
| < 0.4250 | 35.90 | 77.59 | 0.1349 |
| < 0.4350 | 35.90 | 75.86 | 0.1176 |
| < 0.4450 | 38.46 | 74.14 | 0.126 |
| < 0.4550 | 38.46 | 72.41 | 0.1087 |
| < 0.4700 | 41.03 | 72.41 | 0.1344 |
| < 0.4850 | 43.59 | 72.41 | 0.16 |
| < 0.4950 | 46.15 | 72.41 | 0.1856 |
| < 0.5100 | 46.15 | 70.69 | 0.1684 |
| < 0.5350 | 46.15 | 68.97 | 0.1512 |
| < 0.5550 | 46.15 | 67.24 | 0.1339 |
| < 0.5650 | 46.15 | 63.79 | 0.0994 |
| < 0.5750 | 46.15 | 62.07 | 0.0822 |
| < 0.5950 | 48.72 | 60.34 | 0.0906 |
| < 0.6150 | 51.28 | 60.34 | 0.1162 |
| < 0.6300 | 58.97 | 58.62 | 0.1759 |
| < 0.6500 | 61.54 | 56.90 | 0.1844 |
| < 0.6650 | 61.54 | 55.17 | 0.1671 |
| < 0.6900 | 61.54 | 53.45 | 0.1499 |
| < 0.7250 | 61.54 | 51.72 | 0.1326 |
| < 0.7450 | 64.10 | 51.72 | 0.1582 |
| < 0.7600 | 66.67 | 51.72 | 0.1839 |
| < 0.7750 | 66.67 | 50.00 | 0.1667 |
| < 0.7850 | 69.23 | 50.00 | 0.1923 |
| < 0.7950 | 71.79 | 50.00 | 0.2179 |
| < 0.8200 | 74.36 | 50.00 | 0.2436 |
| < 0.8450 | 74.36 | 48.28 | 0.2264 |
| < 0.8550 | 74.36 | 46.55 | 0.2091 |
| < 0.8750 | 74.36 | 44.83 | 0.1919 |
| < 0.8950 | 76.92 | 44.83 | 0.2175 |
| < 0.9050 | 76.92 | 43.10 | 0.2002 |
| < 0.9200 | 79.49 | 43.10 | 0.2259 |
| < 0.9400 | 82.05 | 43.10 | 0.2515 |
| **< 0.9600** | 84.62 | 43.10 | **^b^0.2772 (Maximal)** |
| < 0.9750 | 84.62 | 41.38 | 0.26 |
| < 0.9900 | 84.62 | 39.66 | 0.2428 |
| < 1.005 | 84.62 | 36.21 | 0.2083 |
| < 1.020 | 84.62 | 34.48 | 0.191 |
| < 1.035 | 84.62 | 32.76 | 0.1738 |
| < 1.050 | 84.62 | 31.03 | 0.1565 |
| < 1.075 | 87.18 | 31.03 | 0.1821 |
| < 1.120 | 89.74 | 31.03 | 0.2077 |
| < 1.165 | 89.74 | 29.31 | 0.1905 |
| < 1.200 | 89.74 | 27.59 | 0.1733 |
| < 1.230 | 89.74 | 25.86 | 0.156 |
| < 1.250 | 92.31 | 25.86 | 0.1817 |
| < 1.280 | 92.31 | 24.14 | 0.1645 |
| < 1.305 | 92.31 | 22.41 | 0.1472 |
| < 1.325 | 92.31 | 18.97 | 0.1128 |
| < 1.350 | 94.87 | 15.52 | 0.1039 |
| < 1.370 | 97.44 | 15.52 | 0.1296 |
| < 1.390 | 97.44 | 12.07 | 0.0951 |
| < 1.430 | 97.44 | 8.621 | 0.06061 |
| < 1.495 | 100.0 | 8.621 | 0.08621 |
| < 1.605 | 100.0 | 5.172 | 0.05172 |
| < 1.780 | 100.0 | 3.448 | 0.03448 |
| < 2.030 | 100.0 | 1.724 | 0.01724 |

This table presents the sensitivity, specificity, and Youden’s index of serum PRMT5 at different cutoff values (ng/mL) for distinguishing between study groups. The study population included 97 biological replicates (independent samples from 39 DCM participants and 58 non-DCM participants), with no technical replicates performed for serum PRMT5 detection.

^a^ Statistical analysis was conducted using receiver operating characteristic (ROC) curve analysis to calculate sensitivity, specificity, and Youden’s index (Youden’s index = sensitivity + specificity - 1) for each cutoff value.

^b^ The maximal Youden’s index of 0.2772 was observed at a serum PRMT5 cutoff of < 0.9600 ng/mL, with a corresponding sensitivity of 84.62% and specificity of 43.10%.

**Table S9. Comparison between HFD/STZ group and Ad-vector+HFD/STZ group**

| **Key Indicators** | **Native HFD/STZ** | **HFD/STZ + Ad-vector** | **p value** |
| --- | --- | --- | --- |
| Cardiomyocyte CSA (μm²) | 269.00 ± 12.51 | 266.97 ± 17.69 | 0.823 |
| Collagen deposition (relative) | 1.87 ± 0.36 | 1.98 ± 0.16 | 0.516 |
| LVEF (%) | 47.02 ± 7.47 | 41.64 ± 3.57 | 0.154 |
| LVIDd (mm) | 3.51 ± 0.36 | 3.49 ± 0.21 | 0.944 |
| LVPWd (mm) | 1.20 ± 0.15 | 1.06 ± 0.07 | 0.080 |

**Table S10. Comparison between HG group and HG+vector group**

| **Key Indicators** | **HG+vector** | **HG** | **p value** |
| --- | --- | --- | --- |
| ANF (Fold of average HG+vector) | 1.00 ± 0.07 | 1.11 ± 0.14 | 0.157 |
| BNP (Fold of average HG+vector) | 1.00 ± 0.09 | 0.95 ± 0.11 | 0.476 |
| β-MHC (Fold of average HG+vector) | 1.00 ± 0.07 | 0.96 ± 0.11 | 0.463 |
| ATP5A1 (Fold of average HG+vector) | 1.00 ± 0.14 | 1.11 ± 0.15 | 0.252 |
| mt-ATP6 (Fold of average HG+vector) | 1.00 ± 0.10 | 0.94 ± 0.08 | 0.313 |
| mt-ND1 (Fold of average HG+vector) | 1.00 ± 0.10 | 1.04 ± 0.10 | 0.542 |
| PGC1α (Fold of average HG+vector) | 1.00 ± 0.08 | 1.06 ± 0.17 | 0.474 |
| ATP (μmol/mg protein) | 22.42 ± 16.40 | 28.77 ± 12.69 | 0.510 |
| Complex I (μmol/mg protein) | 40.85 ± 3.75 | 36.16 ± 7.51 | 0.249 |
